# The essential *Rhodobacter sphaeroides* CenKR two-component system regulates cell division and envelope biosynthesis

**DOI:** 10.1371/journal.pgen.1010270

**Published:** 2022-06-29

**Authors:** Bryan D. Lakey, Kevin S. Myers, François Alberge, Erin L. Mettert, Patricia J. Kiley, Daniel R. Noguera, Timothy J. Donohue

**Affiliations:** 1 Wisconsin Energy Institute, Great Lakes Bioenergy Research Center, University of Wisconsin-Madison, Madison, Wisconsin, United States of America; 2 Laboratory of Genetics, University of Wisconsin-Madison, Madison, Wisconsin, United States of America; 3 Department of Biomolecular Chemistry, University of Wisconsin-Madison, Madison, Wisconsin, United States of America; 4 Department of Civil and Environmental Engineering, University of Wisconsin-Madison, Madison, Wisconsin, United States of America; 5 Department of Bacteriology, University of Wisconsin-Madison, Madison, Wisconsin, United States of America; Michigan State University, UNITED STATES

## Abstract

Bacterial two-component systems (TCSs) often function through the detection of an extracytoplasmic stimulus and the transduction of a signal by a transmembrane sensory histidine kinase. This kinase then initiates a series of reversible phosphorylation modifications to regulate the activity of a cognate, cytoplasmic response regulator as a transcription factor. Several TCSs have been implicated in the regulation of cell cycle dynamics, cell envelope integrity, or cell wall development in *Escherichia coli* and other well-studied Gram-negative model organisms. However, many α-proteobacteria lack homologs to these regulators, so an understanding of how α-proteobacteria orchestrate extracytoplasmic events is lacking. In this work we identify an essential TCS, CenKR (**C**ell **en**velope **K**inase and **R**egulator), in the α-proteobacterium *Rhodobacter sphaeroides* and show that modulation of its activity results in major morphological changes. Using genetic and biochemical approaches, we dissect the requirements for the phosphotransfer event between CenK and CenR, use this information to manipulate the activity of this TCS *in vivo*, and identify genes that are directly and indirectly controlled by CenKR in *Rb*. *sphaeroides*. Combining ChIP-seq and RNA-seq, we show that the CenKR TCS plays a direct role in maintenance of the cell envelope, regulates the expression of subunits of the Tol-Pal outer membrane division complex, and indirectly modulates the expression of peptidoglycan biosynthetic genes. CenKR represents the first TCS reported to directly control the expression of Tol-Pal machinery genes in Gram-negative bacteria, and we predict that homologs of this TCS serve a similar function in other closely related organisms. We propose that *Rb*. *sphaeroides* genes of unknown function that are directly regulated by CenKR play unknown roles in cell envelope biosynthesis, assembly, and/or remodeling in this and other α-proteobacteria.

## Author summary

The bacterial cell envelope is home to an array of important functions including energy conservation, motility, influx/efflux of nutrients and toxins, modulation of cell morphology and division, cell-cell interaction, and biofilm formation. Consequently, it is a major target of antibiotics and antimicrobial agents that inhibit these essential processes. Key to the recognition of environmental stressors or stimuli are bacterial TCSs, however systems that monitor or directly regulate cell envelope assembly and homeostasis are not widely conserved amongst bacteria. Here, we use *Rhodobacter sphaeroides* as a model to investigate the function of the CenKR TCS in this and other α-proteobacteria. We show that this essential TCS plays a key role in maintenance of the cell envelope through the regulation of outer membrane integrity and division, cell wall remodeling and homeostasis, and an alternate sigma factor that controls global cellular stress response. We provide evidence that this TCS and its function is widely conserved in α-proteobacteria and identify genes of unknown function as candidates for the study of cell envelope assembly in this and related bacteria.

## Introduction

The Gram-negative cell envelope serves as a selectively permeable barrier to separate and protect a cell from its external environment. This inter-connected compartment is comprised of an inner membrane (IM), the periplasmic space, a thin layer of peptidoglycan (PG), and an outer membrane (OM) [1]. The IM is a phospholipid bilayer separating the cytoplasm from the periplasmic space. Encircling the periplasm is the OM, composed of an inner leaflet of phospholipids and an outer leaflet of protective, charged lipopolysaccharides (LPS) that acts a rigid, semi-permeable barrier [2,3]. Closely associated to the OM is the PG cell wall, a load bearing, mesh-like structure composed of repeating disaccharide units that are cross-linked via short peptide side chains [4,5]. Together, the cell envelope is critical for maintaining cell shape, organizing and facilitating cell division, and protecting cells from deleterious environmental threats such as antibiotics, desiccation, and osmotic stress. Given the many essential functions of the cell envelope, a wide range of naturally occurring toxins, host defense systems, and clinically important antibiotics target cell envelope biosynthetic machinery.

To combat these threats, the Gram-negative cell envelope also serves as a major control center for sensing and rapidly responding to environmental stimuli. Fundamental to this activity are two-component systems (TCSs) which rely on regulated, transmitter-receiver protein communication to monitor cell homeostasis and adjust cell structure, physiology, and behavior in response to internal or external cues [6]. Canonical TCS are comprised of a transmembrane histidine kinase (HK) that upon recognizing an envelope- or extracellular-derived signal autophosphorylates a cytoplasmic C-terminal histidine residue [6–8]. The phosphate of the activated HK is then transferred to its cytoplasmic, cognate response regulator (RR), often resulting in conformational changes and activation of an effector domain to elicit a specific cellular response [6–8]. Commonly, RRs are DNA binding proteins that directly control the transcription of target genes [9]. Several TCSs which regulate envelope functions and mitigate a variety of membrane or cell wall perturbations have been described in Gram-negative bacteria such as *Escherichia coli* Cpx [10–12], Rcs [11–13], and BaeSR [14] and *Caulobacter crescentus* ChvGI [15]. However, these characterized TCS are not generally conserved across bacterial phyla and typically control the transcription of genes whose products are envelope proteases, periplasmic chaperones, function in antibiotic catabolism, or produce alternate PG crosslinks to maintain cell wall integrity [11,16–21]. Moreover, examples of TCSs that directly regulate cell wall metabolism in *Vibrio cholerae* (WigRK [22]) and *Mycobacterium tuberculosis* (MtrAB [23,24]) are specific to each respective bacterial genus and are necessary for the individual pathogenic lifestyles of these bacteria. Therefore, conserved systems that directly regulate the expression of cell envelope biosynthetic genes remain elusive. Previously, Lemmer *et*. *al*. identified a HK (encoded at the *RSP_1056* locus) in the Gram-negative α-proteobacterium *Rhodobacter sphaeroides* that when disrupted, resulted in cell envelope changes that included alterations in shape, increased phospholipid content, and sensitivity to detergents and β-lactam antibiotics that target the cell envelope and wall [25]. Together, these results suggested that RSP_1056 is a *trans*-membrane HK that plays a previously unknown role in cell envelope homeostasis [25].

In this study, we demonstrate the predicted kinase activity of RSP_1056 and identify RSP_0847 as the essential cognate RR for this TCS. We apply a systems biology approach, combining genome-scale chromatin immunoprecipitation followed by high-throughput sequencing (ChIP-seq) and analysis of strain specific changes in global transcript abundance (RNA-seq) to identify genes whose expression are directly and indirectly impacted *in vivo* by genetically altering TCS activity. From these data, we identify the direct targets of RSP_0847 and make predictions for how the altered transcription of these genes lead to the observed phenotypes of strains with changes in activity of this TCS. Based on these data, we propose that the RSP_1056-RSP_0847 TCS, renamed CenK-CenR based on homology with a described TCS in *C*. *crescentus* [26], is henceforth a regulator of cell division, bacterial envelope development and homeostasis, and OM integrity, specifically through the regulation of the Tol complex. Finally, we show that other α-proteobacteria of agricultural, industrial, and medical relevance, lacking well-studied cell envelope development and stress responses, contain homologs of this TCS.

## Results

### RSP_1056 is a histidine kinase that phosphorylates the response regulator RSP_0847

**Fig 1:**
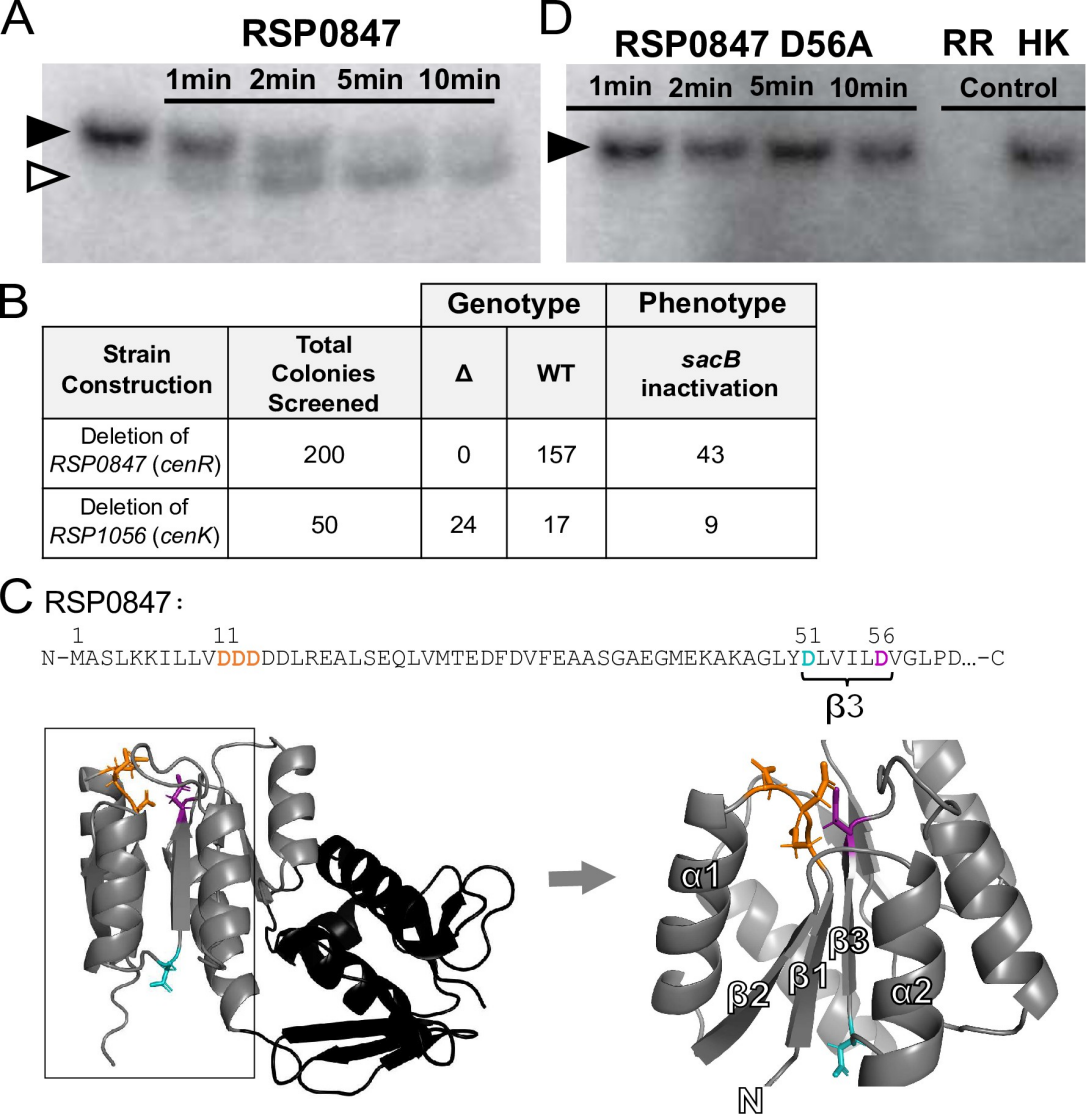
RSP_1056-RSP_0847 comprise an essential TCS. **(A)** RSP_1056 is phosphorylated after incubation with [γ^32^P]-ATP (lane 1). When recombinant response regulator (RR) RSP_0847 was added, phosphotransfer between RSP_1056 (black arrow) and RSP_0847 (white arrow) was detected by the presence of two radiolabeled species. **(B)** Attempts to produce an in-frame, markerless deletion of *RSP_0847* were unsuccessful. Double crossover events were scored (phenotype), and deletions confirmed by PCR (genotype). **(C)** Structural modeling of RSP_0847 using I-TASSER TM-align [34–36]. The RR N-terminus containing the receiver domain (grey) and C-terminal DNA-binding winged helix-turn-helix motif (black) are shown. Predicted Mg^2+^ coordinating aspartate residues between α-helix 1 and β-sheet 1 (orange, D11-D13), aspartate 56 (magenta) on the C-terminal end of β-sheet 3, and aspartate 51 (cyan) on the N-terminal end of β-sheet 3, are labeled in the RR receiver domain (zoomed image). The amino acid sequence of the receiver domain, aspartates of interest, and β3 is shown. **(D)** Recombinant RSP_0847 harboring an alanine substitution at aspartate position 56 (D56A) was tested for *in vitro* phosphotransfer from radiolabeled RSP_1056. Phosphotransfer between radiolabeled RSP_1056 and RSP_0847(D56A) was not detected. Control reactions containing only [γ^32^P]-ATP and RSP_0847 (lane 5) and autophosphorylated RSP_1056 incubated for 10 minutes (lane 6) are shown.

To determine how disruption of *RSP_1056* impacts cellular events [25], we sought to identify the predicted phosphorylation target of RSP_1056. To test that RSP_1056 is indeed a HK, we expressed the predicted cytoplasmic portion of this putative trans-membrane protein in *E*. *coli*. After purification, this recombinant form of a truncated RSP_1056 was incubated with [γ^32^P]-ATP, and a stable autophosphorylated species corresponding to the molecular weight of the truncated RSP_1056 protein was observed (Fig 1A, lane 1), confirming the prediction that RSP_1056 is a kinase.

Genes encoding TCS pairs are often found in the same transcriptional unit. However, genomic neighborhood analysis did not identify any putative RR genes in the vicinity of the gene encoding the RSP_1056 HK. The *Rb*. *sphaeroides* genome encodes 27 predicted RRs that are unpaired with corresponding HK genes that could be phosphoacceptors of RSP_1056. To identify potential candidate RRs for the RSP_1056 kinase, we used a publicly available *in silico* prediction tool (SwissRegulon -TCS [27]) to infer potential HK-RR orphaned pairs based on conserved sequence-specific interactions between protein domains and specific residues that are canonically involved in phosphotransfer by TCS [27–29]. This approach produced three candidate RRs as phosphoacceptors for RSP_1056. One of these, RSP_0847, had a high Bayesian probability (>0.80) as a phosphoaccepting partner for RSP_1056, while two others (RSP_1274 and RSP_1083) had probabilities of 0.14 and 0.05 [27], respectively (S1A Fig). Therefore, we hypothesized that RSP_1056 and RSP_0847 most likely formed a TCS in *Rb*. *sphaeroides*.

To test whether RSP_0847 was the cognate response regulator, we assayed time-dependent *in vitro* phosphotransfer activity from the ^32^P-labeled RSP_1056 to a purified, recombinant form of the predicted RR RSP_0847 *in vitro* (Fig 1A). Detectable phosphate transfer was observed in reactions containing RSP_0847 (Fig 1A, white arrow), with the appearance of radiolabeled RSP_0847 after one minute of incubation (Fig 1A, lane 2). Such rapid phosphotransfer from a HK to a RR is characteristic of an interaction between members of known TCSs, as HKs exhibit strong kinetic preference for their *in vivo* cognate RR target *in vitro* [26,30–32]. Using this same assay, we found that phosphotransfer was not observed between RSP_1056 and the other two candidate RRs (RSP_1083 and RSP_1274, S1B Fig). Together, the rapid and specific *in vitro* phosphotransfer between RSP_1056 and RSP_0847 provides strong support that these two proteins form a TCS in *Rb*. *sphaeroides*.

### The gene encoding the RR RSP_0847 is essential in *Rb*. *sphaeroides*

A query of the NCBI protein database identified the HK CenK (CCNA_00564) and RR CenR (CCNA_03859), **C**ell **en**velope **K**inase and **R**egulator, in the α-proteobacterium *C*. *crescentus* as potential homologs of RSP_1056 (35% amino acid identify, E-value 1e-76) and RSP_0847 (69.5% amino acid identity, E-value 2e-107), respectively. In *C*. *crescentus*, both *cenK* and *cenR* are reported to be essential genes encoded at separate genomic loci, with depletion of either protein resulting in severe OM blebbing and cell lysis through unknown mechanisms [26]. Consistent with the previously proposed essential nature of CenKR, genome-scale Tn-seq analysis of *Rb*. *sphaeroides* (S2A and S2B Fig) predicted that the gene encoding the RR RSP_0847 was essential [33]. However, unlike in *C*. *crescentus*, this analysis identified transposon insertions in *RSP_1056* suggesting this gene is not essential in the conditions tested [25,33].

To independently test if *RSP_0847* is an essential gene, we attempted to construct an in-frame deletion of this gene using the same method that we used to generate an in-frame deletion of *RSP_1056* (Fig 1B). We were unable to recover a strain containing a chromosomal deletion of *RSP_0847*, confirming its essentiality in *Rb*. *sphaeroides*. To independently confirm the functionality of the recombineering plasmid, we attempted to recover a chromosomal deletion of *RSP_0847* in a strain constitutively expressing *RSP_0847* from an ectopic plasmid (S2B and S2E Fig). We were able to efficiently recover in-frame deletions of the genomic copy of *RSP_0847* in this background (S2C Fig) confirming the efficacy of our plasmid to inactivate this locus. Together, these biochemical and genetic analyses provide evidence that the *Rb*. *sphaeroides* RSP_1056-RSP_0847 TCS is likely a homolog of *C*. *crescentus* CenKR [26]. Therefore, we will hereafter refer to *RSP_1056* as *cenK* and *RSP_0847* as *cenR*.

### Aspartate 56 in CenR is critical for phosphorylation by CenK

To further explore the impact and mechanism of phosphotransfer from CenK to CenR, we sought to identify the CenR residue that was required for phosphorylation by CenK. To do this, we used the I-TASSER homology modeling tool [34–36] to predict which CenR aspartate residue acts as a phosphoacceptor from CenK. The receiver domain of OmpR-family RRs, like CenR, contains five-stranded parallel β-sheets flanked by two α-helices on the N-terminus and three α-helices on the other, with the canonical phosphoaccepting aspartate residue located at the end of the third β-sheet [7,8]. This residue is located proximal to negatively charged residues between α1 and β1, which coordinate a Mg^2+^ ion to catalyze phosphorylation [7,8]. The I-TASSER structural model of CenR (Fig 1C) we used was assembled based on similarity to the structure of a RR in *M*. *tuberculosis* (PDB accession number: 1YS6; identity 0.31, coverage 0.97, TM-score 0.87, RMSD 2.16). This model predicts that residue D56 in CenR (Fig 1C, magenta) is proximal to the three aspartate residues, D11-D13, that coordinate Mg^2+^ (Fig 1C, orange), and likely the residue that participates in phosphotransfer from CenK.

To assess if an aspartate at residue D56 is needed for CenR phosphorylation, we expressed and purified a variant RSP_0847 protein containing an alanine substitution (D56A) and tested for activity in a phosphotransfer assay with ^32^P-labeled CenK (Fig 1D). We observed no transfer between ^32^P-labeled CenK and CenR(D56A) under the same conditions where wild-type RR was phosphorylated, demonstrating that an aspartate at residue 56 is necessary for this protein to serve as a phosphoacceptor from phosphorylated CenK. Previously, it had been proposed that *Rb*. *sphaeroides* CenR residue D51 is a site of phosphorylation [37]. However, given the predicted orientation of the CenR D51 side chain away from the catalytic phosphorylation site at the N-terminus of β1, and the inability of CenR(D56A) to be phosphorylated by CenK *in vitro*, we propose that D56 is the site of phosphorylation of this RR (Fig 1C).

### Modulation of the activity of CenR results in cell length defects

Using this *cenR*(D56A) allele, we asked if we could disrupt phosphorylation of CenR *in vivo*. As we were able to delete *cenK* (Fig 1B), we hypothesized that a non-phosphorylatable form of CenR would be sufficient to support cell viability and would phenocopy cells containing the Δ*cenK* allele. To test this hypothesis, we successfully created a strain containing an alanine codon at residue D56 of the native *cenR* locus (*cenR*(D56A)), using the same methods that were unable to produce a strain containing an in-frame deletion of *cenR*.

**Fig 2:**
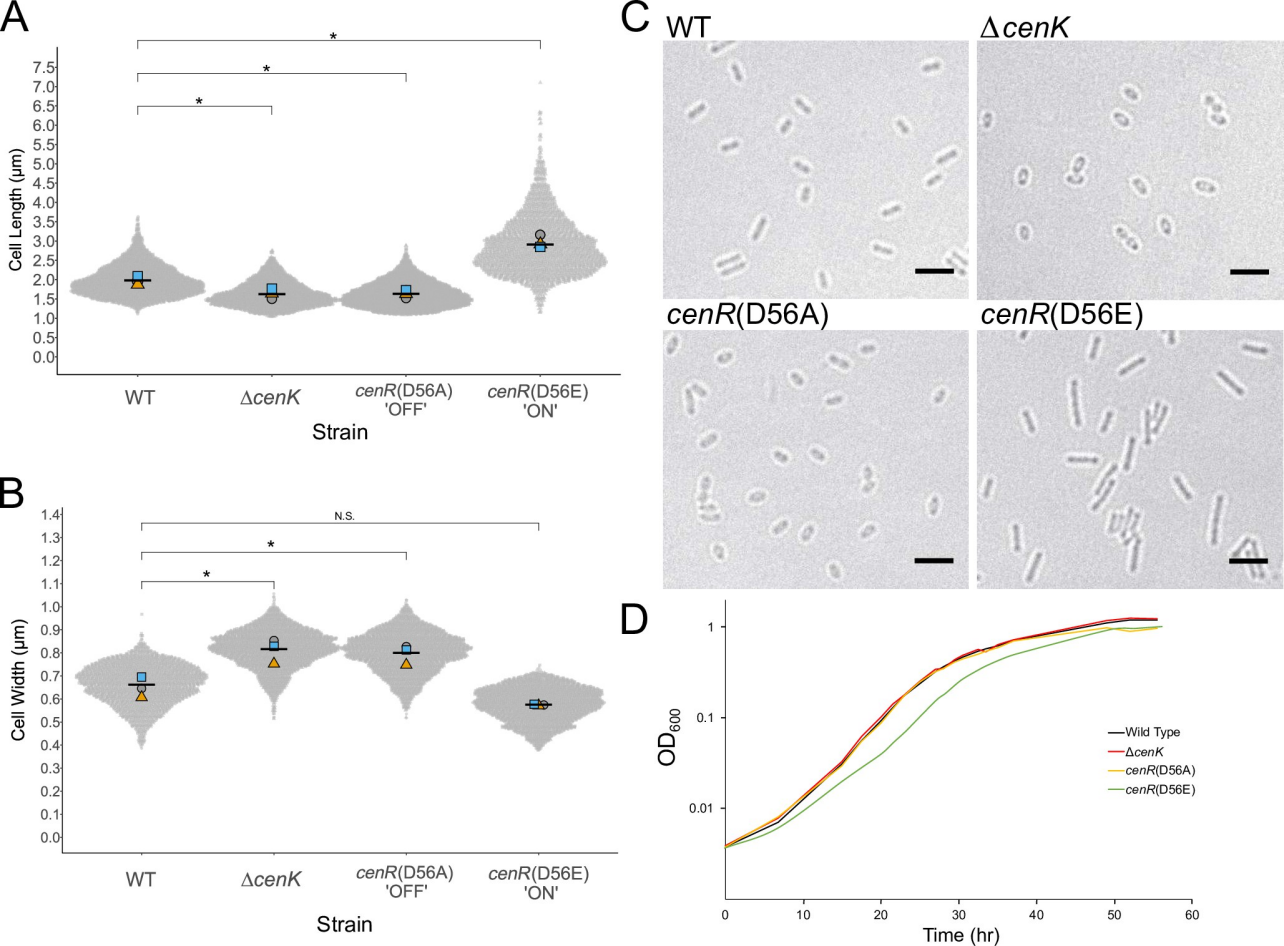
Impact of changes in CenKR activity on cell dimensions. **(Panels A-C)** Bright-field microscopy measurements of the length and width of exponential phase cells displayed as beeswarms plots [135]. Mean values from each of the three biological replicates (grey circles, yellow triangles, and blue squares, respectively) and mean values for each strain are shown (black bar). For each biological replicate, >1000 cells were analyzed. Unpaired t-tests were used to compare pooled cell dimension data from the mean values of each biological replicates (n = 3) [133]. Significant *P* values < 0.05 are indicated by an asterisk. **(A)** Cell length measurements of wild-type (WT) and indicated CenKR mutants: (mean ± SD) WT = 1.98 ± 0.11 μm; Δ*cenK* = 1.62 ± 0.13 μm; *cenR*(D56A) = 1.63 ± 0.10 μm; *cenR*(D56E) = 2.91 ± 0.17 μm (see text for explanation of individual alleles). **(B)** Cell width measurements of WT and indicated CenKR mutants: WT = 0.66 ± 0.04 μm; Δ*cenK* = 0.82 ± 0.05 μm; *cenR*(D56A) = 0.80 ± 0.04 μm; *cenR*(D56E) = 0.57 ± 0.01 μm. **(C)** Bright-field microscopy images of wild-type and indicated CenKR mutant strains (scale bar = 3 μm). **(D)** Growth curve of *Rb*. *sphaeroides* strains. Hyperactivation of CenR results in slower growth rate compared to WT cells (3.5 hours vs 2.5 hour doubling time, respectively).

As previous studies showed that loss of CenK had an observable impact on cell shape [25], we tested the impact of the CenR D56A substitution *in vivo* by measuring cell dimensions using bright-field microscopy. We observed that cells containing the *cenR*(D56A) allele were short (length: WT = 1.98 ± 0.11 μm; *cenR*(D56A) = 1.63 ± 0.10 μm; Δ*cenK* = 1.62 ± 0.13 μm) and more spherical (width: WT = 0.66 ± 0.04 μm; *cenR*(D56A) = 0.80 ± 0.04 μm; Δ*cenK* = 0.82 ± 0.05 μm) in comparison to wild-type cells (Fig 2A–2C). As the *cenR*(D56A) allele phenocopied the features of the Δ*cenK* strain, this supports the conclusion that reduction in activity of this TCS is the cause of the observed changes in cell shape. Furthermore, *Rb*. *sphaeroides* cells containing either the Δ*cenK* or *cenR*(D56A) alleles grew at a similar rate compared to wild-type cells (doubling time: ~2.5 hrs) and reached a similar final cell density (Fig 2D). From our *in vitro* and *in vivo* data, we conclude that the CenR D56A amino acid substitution reduces but does not completely abolish activity of the CenKR TCS.

It has been established that substitution of the phosphoaccepting aspartate residue with a glutamate can sterically mimic phosphorylation of other RRs [38,39]. Therefore, we hypothesized that introduction of the phosphomimetic amino acid change D56E in CenR would result in a cell elongation phenotype. We generated a strain containing a glutamate codon at position 56 of the native *cenR* locus (*cenR*(D56E)), and measured cell dimensions finding that *cenR*(D56E) cells were longer (2.91 ± 0.17 μm) than wild-type cells (Fig 2A and 2C). We also observed that cells containing the *cenR*(D56E) allele grew slower than wild-type cells (*cenR*(D56E) ~3.5 hrs, WT ~ 2.5 hrs, Fig 2D) suggesting that constitutive activity of CenR has pleiotropic effects on growth and/or cell division rates. To test if the impact of the *cenR*(D56E) allele is independent of CenK activity, we introduced this mutation in a Δ*cenK* strain. We observed Δ*cenK cenR*(D56E) also produced longer cells than those containing a wild-type CenR protein (S3 Fig, 2.91 ± 0.06 μm), confirming that the D56E substitution in CenR is sufficient to increase the activity of the RR and change cell length and growth rate.

### Characterization of the genome-wide binding sites of CenR

**Fig 3:**
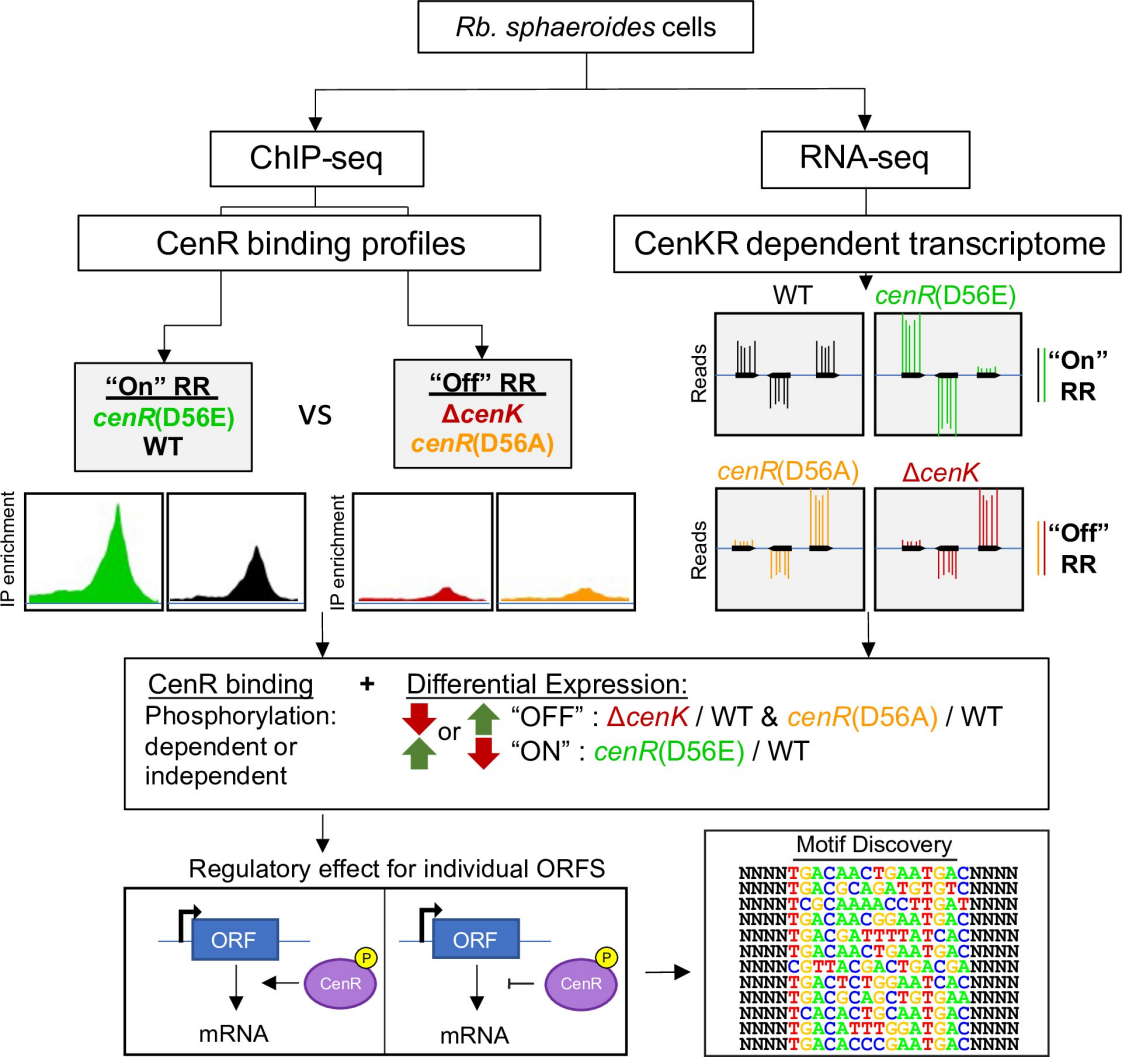
Strategy used to define members of the CenKR regulon. The *in vivo* genome-wide CenR binding sites and CenKR-dependent transcriptomic data were generated from strains with a hyperactive (CenR D56E), wild-type (WT), and low activity (CenR D56A and ΔCenK) TCS. Combined data sets were used to identify genes directly regulated by CenR and the mode of regulation of each candidate gene. Genomic CenR binding sites were then analyzed for a putative DNA binding motif recognized by CenR to further refine the predicted CenR regulon.

To understand how changes in CenKR activity led to the observed cellular changes, we sought to identify genes comprising the direct regulon for this TCS (Fig 3). We expected CenR to bind DNA since it is predicted to be within the OmpR RR family and contains a characteristic winged helix-turn-helix motif in the C-terminal effector domain of the protein (Fig 1C) [6]. To identify potential CenR binding sites, we used ChIP-seq to map the *in vivo* DNA binding by this protein in strains containing wild-type, increased (*cenR*(D56E)), and decreased (Δ*cenK* or *cenR*(D56A)) activity of this TCS. Using the MOSAiCS [40] peak finding algorithm, we identified 458 sites across the *Rb*. *sphaeroides* genome as putative CenR binding sites (S1 Data). Of these, ~60% (273) were present in three or more biological replicates and were located near previously mapped transcription start sites (TSSs) of cells grown under the same conditions used to monitor DNA binding by this RR [41]. The remaining ~40%, found in less than three biological replicates, located in intragenic regions, or between coding regions of convergent genes, were not used in the subsequent analysis.

To identify the CenR binding events which led to changes in gene expression, we used RNA-seq to compare global transcript levels in the same strains used for ChIP-seq experiments (S2 Data). A total of 597 genes were differentially expressed in at least two strains with log_2_-fold change greater than 0.5 or less than -0.5 and a false discovery rate (FDR) of less than 0.05. Based on the impact of CenR amino acid changes at residue D56 on cells, we predicted that genes with increased transcript levels in a strain containing a hyperactive TCS (CenR D56E) should show decreased expression in cells containing reduced CenKR activity (CenR D56A), and vice versa. Of the 597 differentially expressed genes (DEGs) among these data sets, 275 showed opposing expression patterns when comparing strains with increased and decreased CenKR activity relative to wild-type (S2 Data). Combing the ChIP-seq results mapping CenR binding sites and transcriptomic analysis of CenKR dependent changes in gene expression, we identified 59 potential transcriptional units (101 DEGs) that both showed CenR DNA binding and differential transcripts levels between wild-type cells and TCS mutants (S1 Table). Of the 59 potential transcriptional units identified, we predict that CenR activates the expression of 42 (71%), while repressing transcription of the other 17 (29%).

### Identification of a CenR binding motif

**Fig 4:**
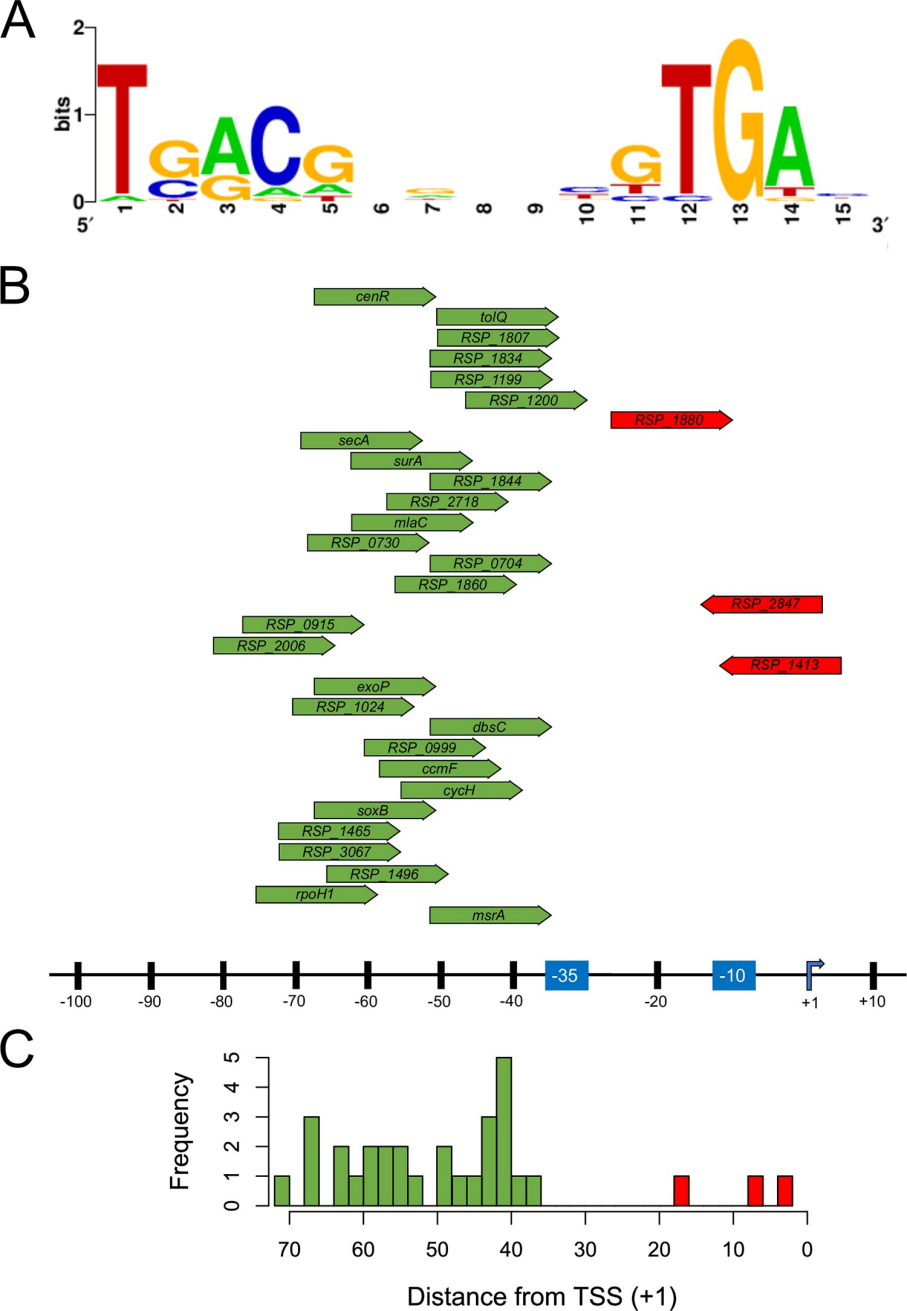
Identification of the CenR DNA binding motif. **(A)** WebLogo [134] representation of CenR consensus sequence determined using the MEME motif finder. Of the 59 promoters identified in ChIP-seq experiments, 31 operons contained the putative CenR consensus sequence TGA-(N8)-TGA. **(B)** Location and orientation of the 31 identified CenR binding sites, in respect to the previously determined TSS [41] and promoter -35 and -10 elements [126] for directly activated (green) and repressed (red) transcription units. The DNA sequence of each binding motif is listed in Table 1. **(C)** Histogram of the distance from the center of each putative CenR binding site and the TSS of the respective transcription unit mapped in (B). Operons predicted to be activated have CenR binding sites upstream of the -35 promoter element, whereas operons predicted to be repressed have CenR binding sites that overlap either the TSS or the predicted promoter elements.

We used MEME [42,43] to identify a conserved DNA sequence logo enriched in regions defined by the 59 ChIP-seq peaks mentioned above. This analysis identified the motif sequence TGA-N_8_-TGA (Fig 4A), a tandem direct repeat within <100 bp upstream of 31 of these 59 putative CenR regulated transcriptional units (S1 Table). Identification of a direct repeat is consistent with other OmpR family transcription factors that often bind DNA as oligomers by recognizing direct repeat sequence motifs [9]. Additionally, the 10 bp distance between the start of each half-site is consistent with that of other known RR that bind B-form DNA [9,44].

We compared the position of each identified CenR binding site to the TSS of each transcriptional unit to make predictions on the role of this RR in controlling expression of these genes (Fig 4C and 4D). Based on this analysis, we found that 28 of the 31 CenR binding sites are located directly upstream of the promoter -35 element (Fig 4C and 4D, green) and oriented co-directionally with the direction of transcription. The location of these predicted CenR binding sites relative to the promoter of these transcription units is consistent with our hypothesis that this RR activates transcription of these genes, likely through interacts with the α subunit of RNA polymerase analogous to other OmpR-family RRs [9]. Of the transcriptional units that we predicted are directly repressed by CenR, the predicted RR binding site is located between the -35 promoter element and known TSS (Fig 4C and 4D, red), with two of the predicted binding sites oriented in the opposite direction of transcription, and one co-directional. This suggests that CenR binding to these sites represses transcription by competing for RNA polymerase binding to promoter elements. Together, we propose that the 31 transcriptional units that contain the conserved TGA-N_8_-TGA motif are members of the CenKR direct regulon (Table 1). While we were unable to find this putative CenR binding site upstream of the other 28 transcriptional units, this does not necessarily mean that these transcriptional units are not direct members of this regulon. However, for this study, we only considered transcriptional units with CenR binding sites in the subsequent analyses.

**Table 1:** The CenKR regulon.

CENTER OF PEAK (bp)^a^	ChIP-seq FOLD ENRICHMENT (IP/INPUT)				DOWNSTREAM GENE			TM Domains^b^	Signal Peptide^c^	Predicted DNA-binding motif: TGA-N8-TGA	TRANSCRIPT FOLD CHANGE (log2)^d^						REGULATORY ROLE^e^	Essential^f^
	D56E	WT	ΔcenK	D56A	ID	GENE NAME	ANNOTATION				cenR(D56E)	FDR	ΔcenK	FDR	cenR(D56A)	FDR		
Cell envelope, transport, and extracytoplasmic targeted proteins																		
2597500	1.4	--	--	--	RSP_0847	*RSP_0847*	two component transcriptional regulator winged helix family	N	N	**ACA**CGAGCGCG**TGA**G	0.49	9.1E-07	-5.16	5.9E-07	-4.34	1.1E-06	+	Y
2413901	4.5	3.2	1.6	1.6	RSP_0672	*tolQ*	Cell division and transport-associated protein TolQ	Y	N	**TGA**CGCAGATG**TGT**T	2.51	2.3E-03	-0.58	6.7E-03	n.s.	n.s.	+	Y
392575	3.8	1.7	--	--	RSP_1807	*RSP_1807*	putative secreted protein	N	Y	**TCG**CAAAACCT**TGA**T	2.72	4.1E-06	-2.04	1.4E-04	-1.97	8.0E-05	+	N
426084	2.8	2.3	--	--	RSP_1834	*RSP_1834*	Beta-lactamase superfamily	N	Y	**TGA**CGCAGCTG**TGA**A	2.47	4.4E-06	-5.56	1.7E-05	-5.43	2.8E-07	+	N
2970115	2.9	2.2	--	--	RSP_1199	*RSP_1199*	L,D-transpeptidases/carboxypeptidases	N	Y	**TGA**CGCGGGCG**TGA**A	2.47	4.4E-05	-1.98	9.9E-05	-1.39	5.1E-04	+	N
2970115*	2.9*	2.2*	--	--	RSP_1200	*RSP_1200*	uncharacterized protein with SCP/PR1 domains	Y	Y	**TGA**GTTGCATT**TGA**A	0.52	1.6E-05	-0.52	3.7E-03	n.s.	n.s.	+	N
478200	2.0	1.6	--	--	RSP_1880	*RSP_1880*	peptidoglycan-binding domain-containing protein	N	Y	**TCA**CATGGCCG**TGT**T	-1.80	4.6E-06	1.24	5.3E-03	n.s.	n.s.	-	N
2933000	1.8	1.4	--	--	RSP_1168	*surA*	PpiC-type peptidyl-prolyl cis-trans isomerase	N	Y	**TGA**CATGATCG**TGT**G	3.40	2.8E-06	-1.48	9.1E-04	-0.74	1.1E-02	+	N
2933000*	1.8*	1.4*	--	--	RSP_1169	*secA*	protein translocase subunit secA	N	N	**TCG**CGCGACGT**TGA**C	1.80	3.0E-06	-0.94	1.7E-03	n.s.	n.s.	+	Y
435330	2.0	--	--	--	RSP_1844	*RSP_1844*	putative periplasmic protein	N	Y	**TGG**CGCAGGCG**TGA**C	1.25	2.2E-05	n.s.	n.s.	n.s.	n.s.	+	N
1368770	2.0	--	--	--	RSP_2718	*RSP_2718*	putative outer membrane protein	Y	Y	**TCA**CCTCCTCG**TGA**G	3.09	8.2E-04	-4.76	5.9E-07	-6.49	2.9E-07	+	N
2641460	1.8	--	--	--	RSP_0890	*mlaC*	Intermembrane phospholipid transport system binding protein	N	Y	**TCA**ATACTTGG**TGA**T	2.47	1.7E-03	-0.74	2.2E-03	-1.14	6.9E-04	+	N
2473818	3.2	1.9	--	--	RSP_0730	*RSP_0730*	TIGR02302 family protein	Y	N	**TCA**CTTCGCCG**TCA**T	2.23	2.0E-04	-1.63	3.7E-04	-0.63	1.5E-02	+	N
2450500	2.0	1.4	--	--	RSP_0704	*RSP_0704*	ABC peptide transporter substrate binding protein	Y	Y	**TCA**CGCAATCG**CAA**C	1.77	2.1E-05	-0.62	4.0E-03	n.s.	n.s.	+	N
458700	1.6	1.7	--	--	RSP_1860	*RSP_1860*	cell wall hydrolase involved in spore germination	N	Y	**TGG**CAAAAATG**TCA**C	1.42	2.1E-03	-0.61	1.1E-02	-1.25	6.6E-04	+	N
1458970	1.6	1.6	--	1.6	RSP_2847	*RSP_2847*	putative lipoprotein (predicted lipid binding domain)	Y	Y	**TCT**TCGTCCCC**TGA**C	-1.20	1.1E-03	0.97	2.7E-02	n.s.	n.s.	-	N
2665900	1.5	--	--	--	RSP_0915	*RSP_0915*	putative periplasmic protein	N	Y	**TGA**CAAACGTG**TTA**G	1.43	5.7E-04	n.s.	n.s.	n.s.	n.s.	+	N
606400	1.6	--	--	--	RSP_2006	*RSP_2006*	uncharacterized protein involved in outer membrane biogenesis	Y	N	**TCA**CGCAATCC**TGA**C	0.67	7.9E-04	n.s.	n.s.	n.s.	n.s.	+	N
930800 (chr2)	1.7	1.6	--	--	RSP_1413	*RSP_1413*	TRAP-T family transporter periplasmic binding component	Y	Y	**TCA**CAAATGCA**TTA**C	-1.34	1.2E-02	0.89	5.8E-02	n.s.	n.s.	-	N
1207306	2.7	1.8	--	1.5	RSP_2561	*exoP*	putative succinoglycan biosynthesis transport protein ExoP	Y	N	**TCA**CGATCCTC**TCA**G	n.s.	n.s.	n.s.	n.s.	-0.60	4.2E-02	+	N
278100	1.7	--	--	--	RSP_1024	*RSP_1024*	Putative MoxR family protein	Y	N	**TCA**CGATCATT**TGT**G	0.85	9.1E-05	-0.56	6.1E-03	-0.84	3.8E-03	+	N
2758300	2.8	1.5	--	--	RSP_1000	*dsbC*	Disulphide bond corrector protein DsbC	N	Y	**TGG**CGCAGTTG**TGA**C	2.48	5.9E-06	-1.64	1.9E-03	-2.07	8.8E-05	+	N
2758300*	2.8*	1.5*	--	--	RSP_0999	*RSP_0999*	putative transcriptional regulator	N	N	**TCG**CGCGACTG**TGG**C	1.37	4.5E-05	n.s.	n.s.	n.s.	n.s.	+	N
1273100	1.6	--	--	--	RSP_2633	*ccmF*	Cytochrome c maturation protein CcmF	Y	N	**TGA**CGGGAAGG**CGA**C	1.06	3.2E-02	-0.21	3.0E-02	n.s.	n.s.	+	Y
1330900	2.2	--	--	--	RSP_2685	*cycH*	Putative cytochrome c-type biogenesis protein cycH	Y	N	**TGA**CGGGATAT**TGA**C	0.79	1.1E-04	n.s.	n.s.	-0.89	1.6E-02	+	Y
1330900*	2.2*	--	--	--	RSP_2686	*soxB*	putative sarcosine oxidase beta subunit	N	N	**TCG**CAACTCGG**TGA**T	3.76	6.0E-05	-1.21	5.5E-04	n.s.	n.s.	+	N
51549	1.9	1.5	--	--	RSP_1465	*RSP_1465*	putative aminoglycoside phosphotransferase	N	N	**TCA**CGCAATCT**TTA**T	0.78	4.5E-05	-1.14	1.2E-03	-1.42	1.6E-03	+	N
106900 (chr2)	1.6	--	--	--	RSP_3067	*RSP_3067*	hypothetical protein	Y	N	**TCG**CAGGATCT**TGA**T	4.51	1.9E-06	n.s.	n.s.	-1.27	8.0E-03	+	N
84880	2.5	1.6	--	--	RSP_1496	*RSP_1496*	Lysozome-like putative lipoprotein	N	Y	**TCG**CGTTTTTC**TGA**A	1.37	1.3E-03	n.s.	n.s.	n.s.	n.s.	+	N
**Cytoplasmic proteins**																		
1039245	4.1	2.5	--	--	RSP_2410	*rpoH1*	RNA polymerase sigma 32 subunit—RpoH	N	N	**TTA**CATTCGCG**TGA**T	3.20	3.2E-04	-3.38	1.4E-06	-2.30	4.6E-06	+	N
2296600	1.6	--	--	--	RSP_0559	*msrA*	Peptide methionine sulfoxide reductase	N	N	**TGG**CGCGAGCG**TGA**T	3.11	8.92–06	-1.78	1.6E-04	-1.41	4.1E-04	+	N

^a^Chromosomal position of ChIP-seq peak (chr2—chromosome 2)

^b^Transmembrane domain predicted using TMHMM 2.0 [45]

^c^Signaling transit peptides identified using SignalP 5.0 [46], scores ≥ 0.6 were interpreted to have predicted transit peptides

^d^log_2_(Fold-Change) are reported relative to WT (i.e. mutant/WT)

^e^Regulatory role of CenR based on changes in gene expresion between TCS mutants relative to WT cells. (+) positively regulated by CenR, (-) negatively regulated by CenK, (NA) Not assigned from data

^f^Essentiality determined previously by Tn-seq analysis [33]

^—^No significant ChIP enrichment

*Peak located between differentially expressed genes

n.s.Not significant, RNA FDR p-value > 0.05 cutoff

To test the role of the TGA-N_8_-TGA direct repeat in CenR binding to promoter regions, we assayed the ability of phosphorylated CenR (CenR~P) to bind to DNA fragments containing the promoter regions of *rpoH1* and *tolQRAB* which are predicted to contain a related sequence (Table 1) and *RSP_2157* which lacks any similarity to this motif. When we assayed binding of CenR~P to each DNA fragment in a gel electromobility shift assay (EMSA), we found that the presence of CenR~P in reactions containing DNA fragments of either *rpoH1* or *tolQRAB* transcriptional units resulted in delayed migration through the gel, relative to reactions containing no protein, which is indicative of DNA-protein interaction (S5 Fig). Furthermore, we observed no change in migration patterns of the DNA fragment of the *RSP_2157* promoter suggesting that the shift in *rpoH1* or *tolQRAB* promoter DNA was dependent on the presence of the TGA-N_8_-TGA direct repeat sequence. The binding patterns observed in this EMSA were similar to what has been previously reported for other OmpR family response regulators, like ArcA, which recognizes a series of three direct repeat motifs [47,48]. Together, this bioinformation and *in vitro* data suggest that this direct repeat is likely the DNA binding motif recognized by CenR *in vivo*.

### CenKR directly activates the expression of genes for envelope stress, remodeling, and cell division

**Fig 5:**
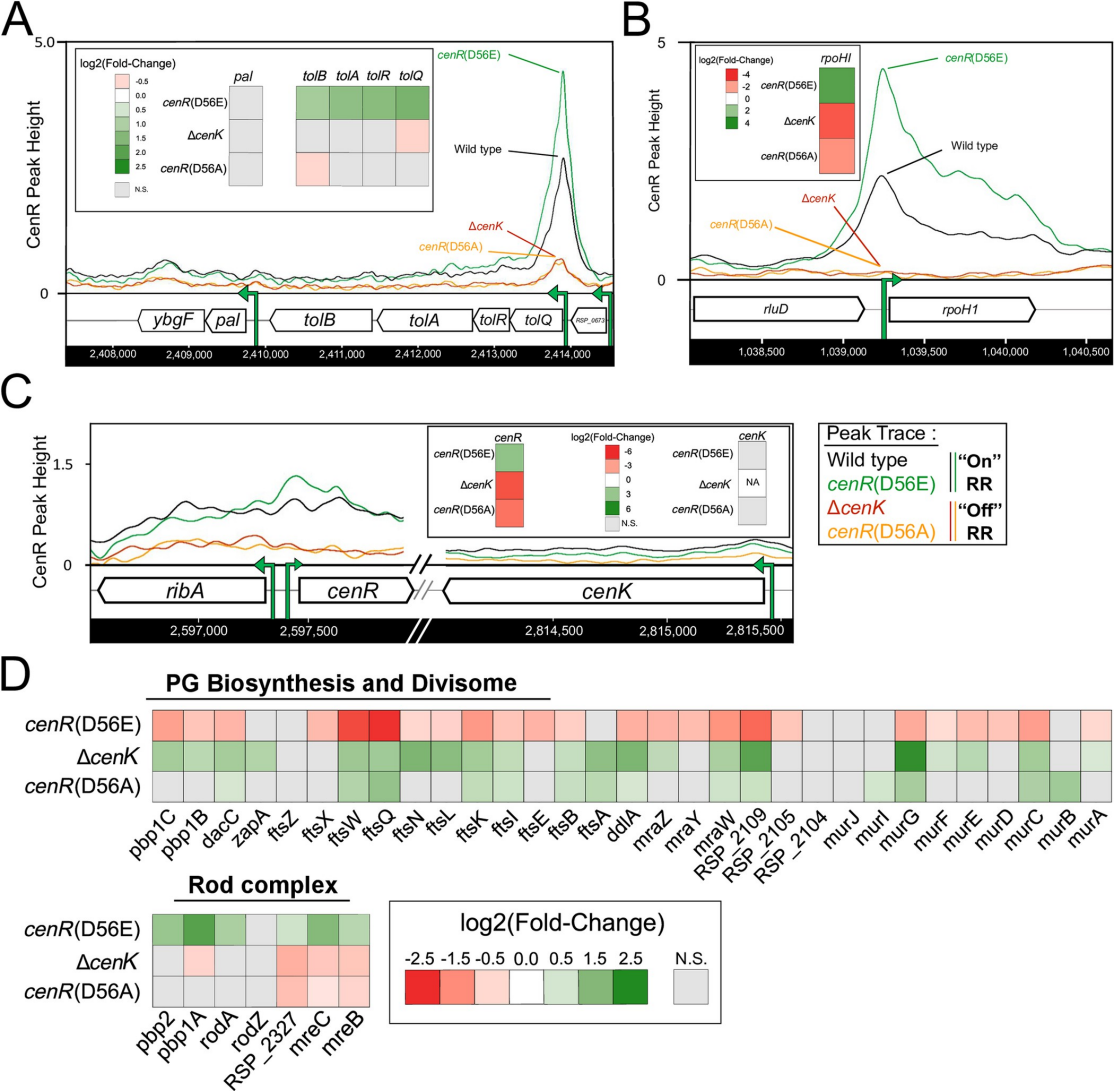
CenKR activity directly regulates the *tolQRAB* operon, the gene encoding the alternate sigma factor *rpoH1*, and indirectly affects the expression of cell wall biosynthesis genes. **(A-C)** Shown are the ChIP-seq data traces of CenR binding upstream of an indicated promoter in TCS “on” strains (*cenR*(D56E) in green, and WT in black) and TCS “low activity” strains (Δ*cenK* in red, and *cenR*(D56A) in orange). ChIP-seq peak heights are represented on the y-axis (fold-enrichment of IP vs input DNA). The chromosomal location is shown on the x-axis with genes represented by arrows pointing in the direction of transcription. TSSs of known promoter(s) are represented as green arrows [41,124]. This inset shows the log_2_ fold change in transcript levels determined from RNA-seq experiments showing change in abundance of the indicated gene in TCS mutant strains relative to WT cells. Non-significant (N.S.) changes in gene expression (FDR > 0.05) are indicated with a grey box **(A)** CenR autoregulates its own expression. CenR binding was detected upstream of *cenR* and increased *cenR* transcripts were found in strains with a hyperactive TCS. No detectable CenR binding was identified upstream of *cenK* and there was no significant change in *cenK* transcript levels in CenR mutants compared to wild-type cells. **(B)** CenR binding enriched upstream of the *tol* operon is predicted to correlate with increased transcript levels. No CenR binding or CenR-dependent change in transcript levels was detected upstream of the operon containing *pal*. **(C)** CenR binding and predicted activation of *rpoHI* expression by this RR. **(D)** Heat map of the effect CenKR TCS activity on expression of PG biosynthesis, division, and elongation machinery. CenKR activity is predicted to indirectly repress PG biosynthesis and cell division related machinery while indirectly activating the expression of the cell elongation machinery (Rod complex).

We used the known or predicted function of the DEGs to gain insight into cellular functions that are directly controlled by CenR. Of the 31 transcriptional units which we predict are direct targets for control by CenR, 29 encode protein products that are targeted to the cell envelope or periplasmic space (Table 1), illustrating this TCS plays a broad role in the function of this cellular compartment. For example, we identified CenR as a direct activator of the transcriptional unit that encodes the Tol proteins of the Tol-Pal complex. CenR enrichment was identified upstream of *tolQ*, the first gene in the *tolQRAB* operon, in all strains analyzed, with decreased occupancy of this RR in Δ*cenK* and *cenR*(D56A) strains (Fig 5A). We found increased abundance of *tolQRAB* transcripts in cells with increased CenKR activity relative to wild-type cells and a slight, yet significant decrease in abundance of the same transcripts in cells with reduced activity of this TCS (Fig 5A).

We found that the expression of other genes known or predicted to function in cell envelope maintenance in other bacteria were predicted to be directly activated by CenR (Table 1). For example, *RSP_1834* encodes a predicted β-lactamase that, in other bacteria, degrades antibiotics that target penicillin binding proteins thereby preventing inhibition of PG synthesis [49], and *RSP_1199* which encodes a putative L,D-transpeptidase that forms crosslinks between peptide sidechains of PG either in response to OM/LPS assembly defects, osmotic stress, or in response to β-lactam antibiotics that inhibit D,D-transpeptidases [5,50,51]. Moreover, *Rb*. *sphaeroides* genes encoding proteins that function in autolysis to digest glycan chains of PG such as *RSP_1496* (a putative lysozyme-like lipoprotein), and *RSP_1860* (a putative cell wall hydrolase) are among those that we predict are directly regulated by CenKR (Table 1). We also identified CenR as a direct transcriptional regulator of the gene encoding the periplasmic retrograde lipid trafficking protein MlaC (RSP_0890), a member of the Mla complex (maintenance of lipid asymmetry) and functions to transport lipids away from the OM [52]. *RSP_0915*, *RSP_1200*, *RSP_1844*, *RSP_2006*, *RSP_2718*, and *RSP_3067* are genes of unknown function that encode proteins targeted to the OM or periplasmic space in other bacteria, suggesting that these predicted members of the direct CenR regulon may have heretofore untested roles in cell envelope biology in *Rb*. *sphaeroides* and other related α-proteobacteria.

This analysis also predicted that several of the gene products in the direct CenR regulon aid in the mitigation of misfolded proteins. These include the homolog of the *E*. *coli* periplasmic chaperon, SurA, which functions to facilitate the folding and assembly of OM proteins [53], and the alternate sigma factor RpoHI (Fig 5B). *Rb*. *sphaeroides* RpoHI directly controls genes encoding protein that are involved in protein homeostasis, maintenance of membrane integrity, and DNA repair [54], and we identified many of these genes as DEGs in our RNA-seq datasets (S2 Data). This latter result suggests that CenKR initiates a global response to general stress. Furthermore, we predict that CenR directly regulates genes that mitigate oxidative and disulfide bond protein stresses *dsbC*, *msr*A [55,56], and cytochrome biogenesis operons *ccmF*-*ccmH* as well as *cycH*. Finally, RRs often regulate their own transcription [9,29]. Consistent with this, we observed CenR binding upstream of *cenR* (Fig 5C), but not upstream of *cenK*. Accordingly, we observed no change in transcription of *cenK*, suggesting expression of *cenK* is not dependent on CenKR activity (Fig 5C).

### Cell wall biosynthetic machinery indirectly responds to changes in CenKR activity

Transcripts from genes encoding PG biosynthesis machinery critical for division and elongation were differentially expressed in cells with altered CenKR activity (Fig 5D). Cells containing the *cenR*(D56E) allele had reduced levels of transcripts from genes whose products are predicted to function in synthesis of cytoplasmic PG precursor molecules (*murB*, *murC*, *murD*, *murE*, *ddlA*, *murF*, *mraY*, and *murG*), their transport across the inner membrane (*ftsW*), and their subsequent assembly into nascent peptide chains (*ftsA*, *ftsQLB*, *ftsK* (*RSP_1495*), *ftsX* (*RSP_0734*), *ftsN* (*RSP_6008*), *pbp1B* (*RSP_0887*), *pbp1C* (*RSP_1355*)) [5,57]. We also found an effect of the *cenR*(D56E) allele on the abundance of transcripts from genes whose products are predicted to function in the divisome (Fig 5D), the complex involved in septal PG synthesis during the latter stages of cell division [5,57]. We found that cells containing the *cenR*(D56E) allele had increased levels of transcripts from genes predicted to encode subunits of the Rod complex (Fig 5D) that synthesizes PG along the lateral cell wall to facilitate cell elongation [5,57]. Combined, these changes in transcript abundance provide a possible explanation for the cell length changes observed in cells that contain the *cenR*(D56E) allele which we predicted increased CenKR TCS activity.

Using ChIP-seq analysis, CenR binding was detected upstream of three (*mraZ*, *ftsW*, *ddlA*) of the seven highly conserved promoters within the division and cell wall biosynthesis (*dcw*) gene cluster (*RSP_2095*-*RSP_2114*) [58], suggesting that this TCS plays a direct role in repressing the transcription of these genes (S1 Table and S6 Fig). However, these promoter regions did not contain the predicted binding motif for this RR in the vicinity of the known TSS, and no decline in CenR enrichment was detected in strains harboring an inactive TCS (S6 Fig). Therefore, we propose that CenR either requires another DNA binding protein to alter transcription, plays an indirect role in the regulation of these genes though hierarchical regulation of other transcription factors, or this represents an artifact in ChIP-seq analysis. In any case, further experiments are required to resolve this. Related to the differential expression of the *dcw* cluster, other genes encoding PG remodeling proteins such as the lytic transglycosylase *mltB* (increased by CenR activity) and D-alanine-D-alanine carboxypeptidase *dacC* (decreased by CenR activity) [5] were predicted to be indirectly regulated by CenR since they were among our list of DEGs in TCS mutants, but lacked any evidence for binding of this RR by ChIP-seq analysis.

### The CenKR TCS is conserved in α-proteobacteria

**Fig 6:**
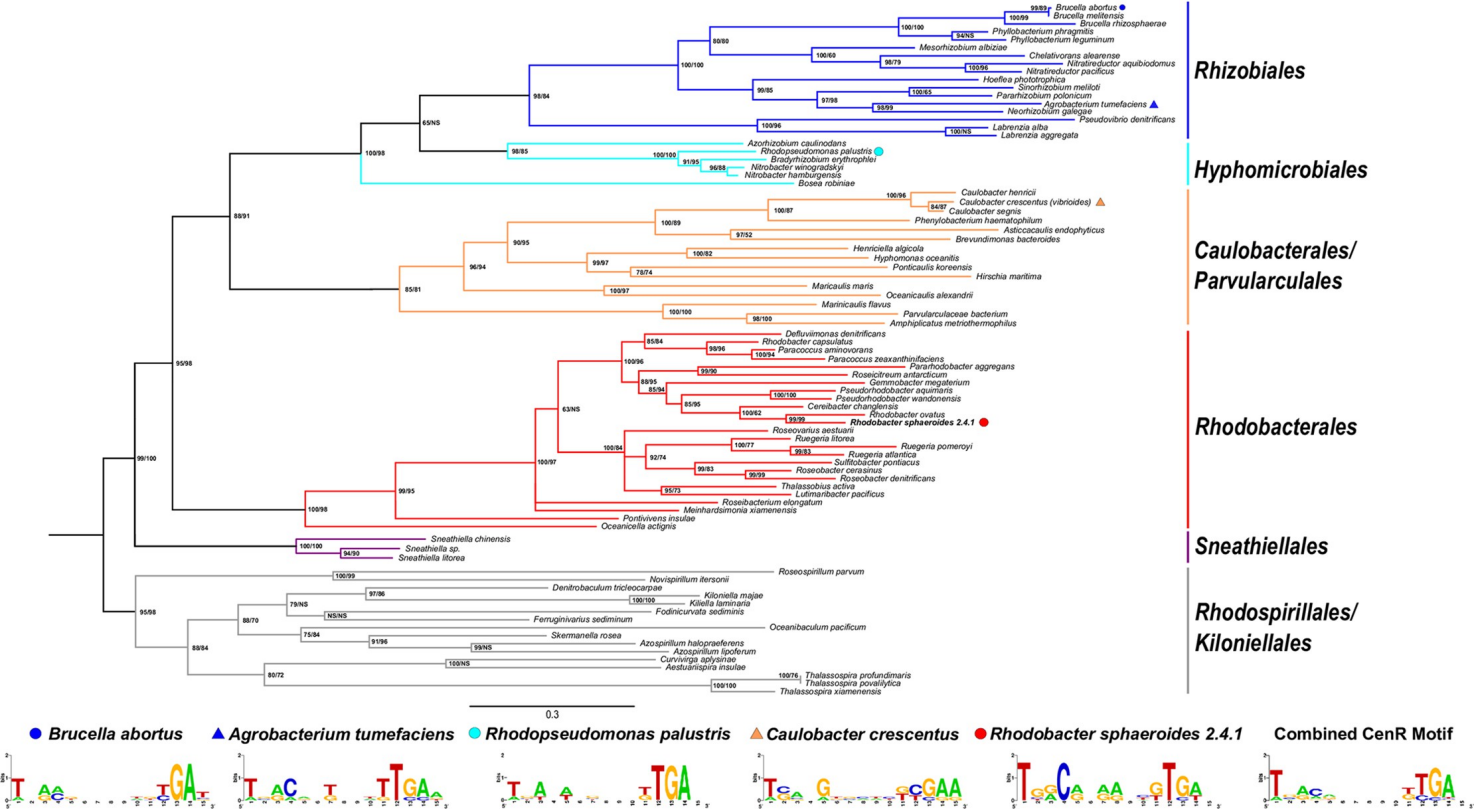
Homologs of CenKR are found in several other α-proteobacteria. **(Phylogeny)** Combined maximum likelihood phylogenetic tree of CenK and CenR identified in α-proteobacteria which had identical topologies. Support values (ML bootstrap) for CenK/CenR are listed for each node. Support values under 50 are labeled NS. **(Bottom panel)** Putative sequence logo of the CenR DNA binding motif derived from analyzing the regions upstream of transcriptional units directly controlled by CenR in indicated α-proteobacteria.

Given the high degree of amino acid sequence similarity between *Rb*. *sphaeroides* and *C*. *crescentus* CenKR proteins and the impact of altered activity of their respective TCSs on cell shape, we sought to determine if aspects of this system were conserved in other bacteria. To probe the conservation of CenKR in other organisms, we constructed separate maximum-likelihood (ML) phylogenies of both CenK and CenR. The topology of both trees was identical and could be combined (Fig 6, node support for CenK/CenR trees, respectively) into a tree that predicts evolutionary relationships agreeing with other α-proteobacteria phylogenies [59–62]. From this analysis, we identified homologs of CenK and CenR within eight orders in the α-proteobacteria phylogeny. These eight formed six, well supported clades consisting of *Rhizobiales* (bootstrap = 98/84), *Hyphomicrobiales* (bs = 98/85) *Caulobacterales*/*Parvularculales* (bs = 85/81), *Rhodobacterales* (bs = 100/98), *Sneathiellales* (bs = 100/100), and *Rhodospirillales*/*Kiloniellales* (bs = 95/98) representing the *Caulobacteridae* subclass [61,62]. Homologs of CenK or CenR were not identified in organisms belonging to the *Sphingomonadales* (subclass *Caulobacteridae*), orders with in the *Rickettsiidae* subclass (encompassing the *Rickettsiales*, *Pelagibacterales*, and *Holosporales* orders), or the *Magnetococcales* at the base of the α-proteobacterial phylogeny [59–62], in members of other proteobacterial groups, or elsewhere in the bacterial phylogeny.

The conservation of CenKR homologs in other α-proteobacteria, as well as the proposed conserved role of the TCS in cell envelope regulation in *C*. *crescentus* [26] leads us to hypothesize that the genes we identified as direct members of this regulon might also be evolutionarily conserved in other related bacteria. To test this hypothesis, we sought to identify predicted CenR DNA binding sites in the genome of other well-studied organisms such as *Agrobacterium tumerfaciens* (*Rhizobiales*), *Brucella aborus* (*Rhizobiales*), *C*. *crescentus* (*Caulobacterales*), and *Rhodopseudomonas palustris* (*Hyphomicrobiales*) that contain this TCS. To do so, we compared the mapped CenR binding sites upstream of *cenR*, *tolQ*, *RSP_1860*, *RSP_1807*, *surA*, and *msrA* (Table 1) in *Rb*. *sphaeroides*, with regions 300 bp upstream of each of these operons in these four related species (S4 Table). This analysis identified the conserved direct repeat TGA-N_8_-TGA upstream of each operon in each analyzed species (Fig 6, bottom panel), supporting the hypothesis that CenKR plays a conserved role in cell envelope regulation in α-proteobacteria.

## Discussion

**Fig 7:**
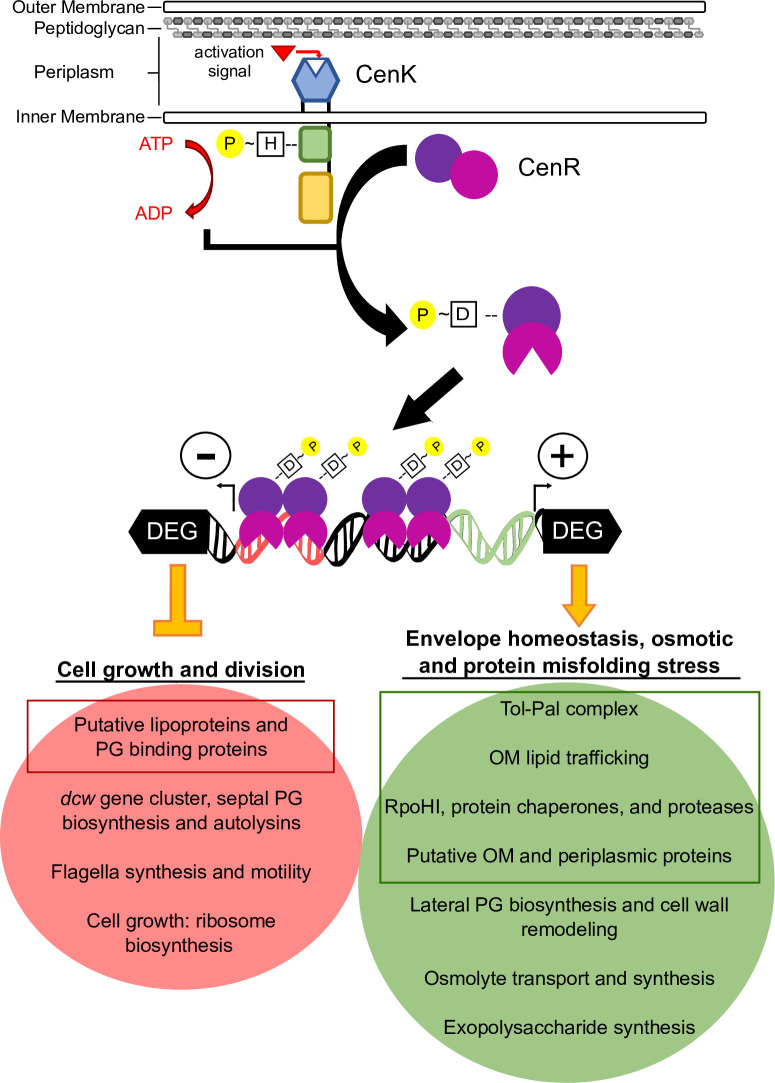
Model for the cellular impacts of direct and indirect CenKR transcriptional regulation. CenK autophosphorylation occurs at a conserved histidine residue within its cytoplasmic domain in response to an unknown signal. CenK then phosphorylates CenR at aspartate 56 within the receiver domain, resulting in conformational changes that allow CenR to bind the promoter regions of direct target genes, repressing transcription by binding within the promoter elements (red) or activating transcription by binding upstream of promoter elements (green). Phosphorylated CenR directly upregulates (green box) genes and pathways that function in the mitigation of cellular or cell envelope stresses, maintenance of the envelope, the Tol complex that plays a vital role in OM division and stability, and other genes of unknown function predicted to be involved in the assembly or repair of cell envelope components. Indirectly, increased activity of CenR results in the increased expression of genes that function in an osmotic stress response and synthesis/remodeling of lateral cell wall (green oval). Phosphorylated CenR directly reduces transcription (red box) of gene products of unknown function that are predicted to be targeted to the cell envelope. Indirectly, increased activity of CenR decreases expression of genes within the *dcw* gene cluster that function in septal PG and cell wall biosynthesis, and genes whose products cleave PG, synthesize flagellar, and are vital for cell growth such as ribosome biosynthesis (red oval).

This work sought to increase our understanding of bacterial cell envelope assembly as well as modifications to this essential cellular compartment. Little is known about these processes in α-proteobacteria since these organisms lack well-studied regulators of cell envelope processes that are known in other proteobacteria. We showed that the α-proteobacterium *Rb*. *sphaeroides* protein RSP_1056 (CenK) is a HK that phosphorylates an OmpR-family RR, RSP_0847 (CenR), and these two proteins comprise a TCS with a high degree of amino acid similarity to *C*. *crescentus* CenKR. We combined genome-scale ChIP-seq with transcript abundance data from cells predicted to have different levels of CenKR activity to identify the direct CenR targets and make predict how this TCS impacts cell envelope functions in this and other α-proteobacteria (Fig 7). Below we highlight new information provided by our analysis of the *Rb*. *sphaeroides* CenKR TCS.

### CenKR comprise an essential TCS in *Rb*. *sphaeroides*

It is known that genes encoding CenK and CenR are essential, encoded at separate loci, and comprise a TCS in *C*. *crescentus* [26]. In contrast, insertions in *Rb*. *sphaeroides cenK* (*RSP_1056*) were identified in two separate transposon insertion libraries, suggesting that unlike in *C*. *crescentus*, this gene is not essential in this α-proteobacterium [33]. However, the saturating genome-wide transposon library that identified *cenK*, did not contain insertions in *cenR* suggesting that this predicted RR is essential in *Rb*. *sphaeroides* (S2 Fig) [25]. Similarly, a Tn-seq screen performed in α-proteobacterium *Rhodopseudomonas palustris* (Fig 6, *Hyphomicrobiales*) identified insertions in the homolog of *cenK* (*RPA0635*), but not *cenR* (*RPA0283*) suggesting that this RR is also essential in this bacterium [63]. To date, only a few bacterial TCSs are known to be essential. Indeed, systematic disruption of the 106 TCS genes in *C*. *crescentus* identified only four essential TCS pairs. Three of these, CenK-CenR, CtrA-CckA, and DivK-DivJ, are known or predicted to be involved in cell cycle or cell envelope processes [26]. Another essential *C*. *crescentus* gene identified in this study was *ntrX*, encoding the RR in the NtrYX TCS that regulates LPS, exopolysaccharide synthesis, and cell wall composition in *Rb*. *sphaeroides* [64]. Other essential TCSs that directly regulate crucial cell wall biosynthesis machinery include WalKR in the Gram-positive organisms *Streptococcus pneumoniae* (also known as VicKR) [65] and *Bacillus subtilis* [66], WigRK in the pathogenic Gram-negative bacterium *V*. *cholerae* [22], and MtrAB in the pathogenic bacterium *Mycobacterium tuberculosis* [23,24]. In contrast, the cell envelope stress response TCSs Cpx and Rcs in Gram-negative γ-proteobacteria like *E*. *coli* are not essential and regulate genes that mitigate OM/LPS, OM protein, or lipoprotein stress rather than directly regulating cell envelope biogenesis machinery [11,67].

*C*. *crescentus ntrX*, *M*. *tuberculosis mtrA*, *S*. *pneumoniae vicR* (*walR*), and *Rb*. *sphaeroides*/*C*. *crescentus cenR* each are examples of essential RRs linked to non-essential HKs, suggesting that loss of the HK reduces TCS activity and that these RRs have some critical function in the absence of phosphorylation [24,26,65]. Supporting this, our *in vivo* analysis of a *cenR*(D56A) allele that abolishes phosphotransfer between CenK and CenR *in vitro* (Fig 1D) suggests that this mutation diminishes CenR activity as the *cenR*(D56A) allele phenocopies the Δ*cenK* strain (Fig 2). Furthermore, in strains with low CenKR activity (Δ*cenK* and *cenR*(D56A)), we detected CenR binding to some promoters by ChIP-seq (Fig 5A). Based on these observations, we propose that CenR has basal activity in the absence of phosphorylation that is sufficient to promote transcription of essential genes and/or pathways, comparable to other TCS [32,65].

### *Rb*. *sphaeroides* CenKR regulates essential division, cell wall, and other envelope processes

Genetic and biochemical characterization of *Rb*. *sphaeroides* CenKR allowed us to identify direct and indirect targets of this TCS. Our data predict that most members of the direct CenR regulon encode proteins that contain signal peptides and are predicted to be targeted to the OM or periplasmic space. These direct CenKR regulon members prominently included predicted OM structural or transport proteins, PG binding proteins, and periplasmic chaperones or proteases. Of the genes predicted to be direct targets of CenR, ~42% are annotated as either hypothetical proteins or have generic descriptions of their function, thus we propose these represent candidates for the future study of envelope components that are controlled by the CenKR TCS. For example, α-proteobacteria lack homologs of the well-studied spatial regulators of *E*. *coli* PG biosynthesis LpoA and LpoB [57,68], so it is possible that one or more members of the CenKR direct regulon provide this function. Our data also predict that CenR indirectly regulates genes encoding PG biosynthesis enzymes that promote lateral cell wall synthesis (Fig 5D). This, plus the observed changes in cell shape when CenKR activity is increased or decreased (Fig 2A–2C) make testable predictions that one or more direct, or indirect, targets of CenKR may have a role in the spatial regulation of cell wall synthesis. Given the relatively thin cell wall of Gram-negative bacteria, precise control of PG synthesis, turnover, and repair is essential, and in other bacterial cells, redundant pathways exist to maintain cell wall integrity in response to environmental signals and membrane-active agents [5]. Furthermore, the number of PG cleavage or crosslinking enzymes (amidases, lytic transglycosylases, carboxypeptidases, and D,D-endopeptidases or D,D/L,D-transpeptidases and glycosyltransferases) vary among bacteria [50,51]. *Rb*. *sphaeroides* contains nine predicted lytic transglycosylases and nine predicted L,D-transpeptidases compared to the eight and six, respectively, that have been identified in *E*. *coli* [50,51]. Our datasets identify *Rb*. *sphaeroides* candidates for gene products that may fill some of these roles or occupy other cellular niches including *RSP_1199*, annotated to encode a predicted L,D-transpeptidase (upregulated by CenKR), and *RSP_1880*, encoding a putative PG binding protein that may have autolytic activity since it is downregulated by CenKR. Thus, we predict that analyzing genes that are direct or indirect CenKR targets will shed new light on the process and control of PG biosynthesis across α-proteobacteria.

We also identified CenR as a direct regulator of the Tol subunits of the Tol-Pal complex (Fig 5A), a system that has been implicated in envelope processes such as OM migration during septal constriction [69,70], coordination of PG remodeling/cleavage at the site of cell division [71,72], and polar localization of proteins [73]. It has also been proposed that Tol-Pal is important for maintaining noncovalent OM-PG interactions in Gram-negative bacteria like *Rb*. *sphaeroides* that lack a homolog of the Braun’s lipoprotein [74]. Recently, it has been shown that the function of Braun’s lipoprotein in α-proteobacteria is facilitated by other OM β-barrel proteins that are covalently bound to PG through the activity of L,D-transpeptidases in α-proteobacteria [74]. Given the effects of disruption of Tol-Pal on OM integrity, leakiness, and OM vesicle production in other organisms [75], we propose that Tol-Pal, or Tol-Pal associated proteins, may coordinate one or more of these process in α-proteobacteria. In other bacteria, a reduction or loss of Tol-Pal components also increases cell permeability and OM fluidity due to an increase in OM phospholipid and a reduction or loss of LPS [75,76]. This also appears to be the case in *Rb*. *sphaeroides* since disruption of *cenK* resulted in increased cellular phospholipids, lipid secretion, and enhanced sensitivity to detergents (like SDS and polymyxin B) which are normally excluded by LPS [25]. We also found that cells containing a phosphomimetic *cenR* allele, *cenR*(D56E), that is predicted to increase activity of this TCS *in vivo*, resulted in cell filamentation, suggesting that CenKR activity coordinates cell division and/or cytokinesis.

It is known that *tol* operon genes encoding IM subunits of the Tol complex are essential in *Rb*. *sphaeroides*, *C*. *crescentus*, and other α-proteobacteria [73,77]. In other bacteria where the Tol system has been well-studied, the IM Tol complex (TolQRA) facilitates Pal localization and PG binding at the septum during cell division, by regulating the interaction between the OM lipoprotein Pal and the periplasmic TolB protein [70,78]. In *E*. *coli* and related organisms, *tolQRA* and *tolB-pal-cpoB* make up two transcriptional units [79]. However, in *Rb*. *sphaeroides*, *C*. *crescentus*, and other α-proteobacteria *tolB* is part of the *tolQRAB* transcriptional unit, and *pal-cpoB* are in a separate transcriptional unit [41,73]. Our analysis indicates that CenR acts directly at *tolQRAB* but, does not directly or indirectly regulate the expression of *pal*-*cpoB* (Fig 5A). As the function of Pal and TolQRAB are proposed to be essential in several α-proteobacteria [33,63,73,75], we propose that organisms with the same *tol*-*pal* synteny rely on CenKR to regulate the activity of Tol-Pal by direct control of *tolQRAB* transcription. Such a mechanism would allow for the regulation of essential Tol driven cell division processes while allowing Pal to maintain other functions independent of the subunits of Tol.

The many roles of Tol-Pal complex as well as the essential role of this machine in several α-proteobacteria [73,75,77] likely contributes to CenR essentiality and further supports our hypothesis that CenKR regulates functions necessary for cell division. To our knowledge, CenKR is the first TCS in Gram-negative bacteria that directly regulates genes encoding Tol complex proteins. Mutations that impact the *E*. *coli* Rcs are reported to increase expression of the Tol-Pal genes [80], and inactivation of the *C*. *crescentus* ChvGI altered expression of genes encoding subunits of the Tol-Pal complex [15]. However, it is unclear in these organisms if these TCSs directly or indirectly regulate the expression of these genes.

### The CenKR TCS also controls cellular stress responses

We identified CenR as a direct activator of *rpoHI* expression (Fig 5B), one of two *Rb*. *sphaeroides* σ^32^ alternate sigma factors, that control transcription of gene products that function in protein quality control and turnover, ion transport, and oxidative stress responses (i.e. disulfide bond and peroxide reduction) [54,81]. Previously, it was proposed that CenR negatively regulates *rpoHI* expression [54]. However, we found reduced levels of transcripts of both *rpoHI* and known RpoHI regulon members in cells containing low CenKR activity (Δ*cenK* or *cenR*(D56A)) and increased levels of these transcripts in cells containing increased activity of this TCS (*cenR*(D56E)). Direct activation of *rpoHI* transcription by CenR suggests that CenKR activates expression, in a hierarchical fashion, of a large set of genes that encode proteins functioning to mitigate general, cytoplasmic, or membrane stress responses.

The *E*. *coli* RseA-σ^E^ system is another master regulator of both unique genes encoding proteins with cell envelope functions and some genes which also are members of the Cpx or Rcs regulons, thereby functioning broadly to monitor extracytoplasmic homeostasis [11,67]. In *E*. *coli* it is proposed that accumulation of unfolded or mislocalized OM proteins activates proteases that degrade the anti-σ^E^ factor RseA, allowing σ^E^ to promote transcription of envelope stress response genes [78]. *Rb*. *sphaeroides* lacks homologs of *E*. *coli* RseA-σ^E^, but it is known that RpoHI is a direct regulator of *cenR* transcription in response to heat shock, oxidative and/or membrane stress signals [54,82]. Thus, we propose the existence of a positive CenR-RpoHI feedback loop that allows a robust, synergistic response to agents that act at the cell envelope. Given the lack of homologs of *E*. *coli* Cps, Rcs, and RseA-σ^E^ in *Rb*. *sphaeroides* and other α-proteobacteria, it is likely that CenKR is a major contributor to a transcriptional response to envelope derived signals.

Other likely indirect effects of changes in CenKR activity include repression of genes involved in chemotaxis, motility, flagellar machinery, and those encoding ribosomal proteins, the latter is consistent with the slow growth rate of strains harboring the hyperactive CenR(D56E) protein (S2 Data). Low CenKR activity in Δ*cenK* or *cenR*(D56A) is also associated with an increase in transcripts from genes encoding proteins that function in polysaccharide production, sarcosine/glycine biosynthesis, and transport of osmolytes which, together, mitigate osmotic stress within the cell [83] and the periplasmic space [84]. Given the role of the OM LPS and the cell wall in resistance to turgor pressure [3] and the altered levels of transcripts from genes whose products are predicted to function in assembly or maintenance of these structures in cells with low CenKR activity, we propose this results in the responsive expression of these osmotic stress response genes. Together, the CenKR TCS is a heretofore unknown regulator of cell envelope changes necessary to combat internal/external turgor pressures.

### The presence and role of CenKR is predicted to be conserved across α-proteobacteria

We identified homologs of CenK and CenR within the *Caulobacteridae* sub-class of α-proteobacteria, including free-living, commensal, and pathogenic organisms. Many of the α-proteobacteria that contain CenKR homologs undergo morphological changes throughout their lifecycle. Purple non-sulfur bacteria like *Rb*. *sphaeroides* and *Rp*. *palustris*, under low oxygen conditions, produce intracytoplasmic membranes as cell envelope invaginations that bud inward from the IM and house the machinery for the light reactions of photosynthesis [85,86]. *C*. *crescentus* and *Rp*. *palustris*, and other members of the *Rhizobiales* that contain CenKR homologs, have a dimorphic life cycle that requires changes in cell envelope, loss of flagella, and the formation of stalks that differentiate free-living swarmer cells to an anchored sessile form [87,88]. The animal parasite *B*. *abortus* relies on membrane changes for macrophage internalization and pathogenesis [89], and the plant pathogen *A*. *tumefaciens*, as well as other members of the *Rhizobiales*, exhibit polar growth and utilize polar budding for division and exhibits a range of morphologies when cell division is altered [90]. These examples showcase some of the cell envelope and cell cycle alterations that are characteristic of α-proteobacteria containing CenKR homologs. We propose that these lifestyles are one reason why these organisms contain cell envelope TCSs and stress response machinery different from those classically studied in *E*. *coli* and other bacteria. We note that we were unable to identify homologues of CenKR in the *Sphingomonadales* or *Rickettsiales* clades (Fig 6), α-proteobacteria that are known to exhibit unusual cell envelope features. *Sphingomonades* contain sphingolipids in the outer leaflet of the OM rather than LPS [91], and members of the *Rickettsiales* include many obligate parasitic bacteria that lack complete cell wall biosynthetic pathways or known membrane-bound enzymes to polymerize PG precursor molecules into the nascent cell wall [92,93]. The existence of CenKR across the *Caulobacteridae* subclass of α-proteobacteria supports a hypothesis that this TCS coordinates a myriad of activities that reside in the OM, LPS, or PG cell wall. There is no recognizable ligand-binding domain in the N-terminal periplasmic or IM region of the CenK HK, so further experiments are needed to identify the signal(s) that controls CenK kinase activity to determine if CenKR responds to signals derived from these cell envelope components.

In closing, we have shown that CenKR is one of several identified TCSs that regulates cell envelope homeostasis in α-proteobacteria [15,64]. However, the function(s) of these other recently identified cell envelope regulators are not necessarily conserved across α-proteobacteria. For example, NtrYX has been implicated as a regulator of root nodule formations that are involved in nitrogen fixations, carbon/nitrogen metabolism, transport systems [94–96], regulation of anaerobic processes [95,97,98], as well as exopolysaccharide biosynthesis, cell envelope composition, and growth [15,64,95,99]. Similarly, in some α-proteobacteria, ChvIG has been linked to growth [15], pathogenesis [100,101], motility [102], and pH sensitivity [103]. In contrast, the presence of a putative *Rb*. *sphaeroides* CenR binding motif (TGA-N_8_-TGA), upstream of several conserved transcriptional units across α-proteobacteria (including the *tol* operon), suggests a similar role for the CenKR in these species. While further study of the signals that control CenKR kinase and DNA binding activity are required to better understand the role of this TCS, we have shown that CenKR is an essential TCS, defined the regulon controlled by this regulatory system, and we predict that it plays a conserved and central role in envelope, cell division, and other processes that are crucial to viability in *Rb*. *sphaeroides* and other α-proteobacteria.

## Materials and methods

### Bacterial strains and growth conditions

*Rb*. *sphaeroides* strains (S2 Table) were grown in Sistrom’s (SIS) minimal medium [104]. Unless specified, aerobic cultures of 10 mL were grown in 125 mL flasks with shaking at 200 rpm, at 30°C for ~18 hours until cells reached an OD_600_ of ~0.5. *Escherichia coli* strains were grown at 37°C in Luria-Bertani medium. As needed, media was supplemented with 50 μg/mL kanamycin, 25 μg/mL spectinomycin, or 10 μM isopropyl β- d-1-thiogalactopyranoside (IPTG).

### Strain construction

All strains, primers, and plasmids used in this study are listed in S2A and S2B Table). In-frame, markerless deletion of *RSP_1056* and allelic exchange within *RSP_0847* was performed via the integration of the suicide vector pk18mobsacB [105]. The genomic regions corresponding to ~1 Kb immediately upstream of the start and downstream of the stop codons of each gene was PCR amplified using Herculase II Fusion DNA Polymerase (Agilent). These PCR products were assembled into PCR linearized pk18mobsacB via Gibson Assembly (New England BioLabs), transformed in DH5α cells (New England BioLabs), and screened by colony PCR of the cloning site and sequenced. Assembled plasmids were mobilized into *Rb*. *sphaeroides* via conjugal mating with *E*. *coli* S17-1 [106]. Single crossover transconjugants were selected for by kanamycin resistance followed by sucrose counter-selection to induce double crossovers. Strains with the desired double crossover were identified by isolation of colonies sensitive to kanamycin and resistant to sucrose (S2A Fig). The locus-specific genotype of each strain was confirmed by colony PCR of chromosomal loci and sequenced with specific primers (S2 Table). Construction of pIND5_spec_-*cenR* was achieved by co-transformation of PCR amplified *cenR* and linearized pIND5_spec_ into *E*. *coli* DH5α (New England BioLabs). Assembled plasmids were mobilized into *Rb*. *sphaeroides* via conjugal mating with *E*. *coli* S17-1 [106]. Markerless deletion of *RSP_0847* was attempted in the presence of pIND5_sepc_-*cenR* as described above except in the presence of spectinomycin antibiotic and IPTG through the selections.

### Microscopy

All strains were grown as described above to an OD_600_ ~ 0.5. Images were acquired on an EVOS FL microscope with 100X oil immersion plan apochromat objective (numerical aperture, 1.40). To facilitate cell segmentation, all bright-field pictures were treated in FIJI [107] with the same process: bandpass filter (large filter, 40 pixels (pxs); small filter, 2 pxs), background subtraction (rolling ball radius = 20 pxs), and contrast enhancing with normalization (0.1%). Segmentation was performed using the plugin MicrobeJ [108] and cell segmentation errors were removed. Cell shape parameters were extracted (S3 Data), figures and statistical analysis were performed in Rstudio using ggplot2 package [109].

### Protein purification and RSP_0847 (CenR) antibody production

The cytosolic domains of the RSP_1056 (CenK) coding sequence (truncated after the second predicted transmembrane helix at residue M195) was PCR amplified and ligated into linearized pVP302K [110] via HiFi Gibson Assembly (New England BioLabs), generating an N-terminal His_8_-tagged protein containing a TEV protease cleave site (H_8_-TEV-HK_cyto_). Candidate RR genes (RSP_0847, RSP_1083, RSP_1247) were ligated into the same expression vector containing a C-terminal His_8_ tag and TEV cleavage site (RR-TEV-H_8_). Candidate TCS expression plasmids were transformed into B834 *E*. *coli* harboring pRARE2 (Novagen) [111,112]. Cells were grown in 500 mL of ZMS-80155 auto-inducing media supplemented with 10 μg/mL kanamycin and 20 μg/mL chloramphenicol for ~24 hours with shaking at room temperature. Cells were harvested by centrifugation, resuspended in 10 mL of lysis buffer (20 mM HEPES-KOH [pH 7.5], 0.5 M NaCl, 5 mM imidazole, 1% glycerol, 1 mM TCEP, 0.1% Triton X-100) and 100 μL of HALT Protease Inhibitor (Thermo), lysed by sonication, and centrifuged at 4°C for 30 min at 20,000 x *g* to generate a lysate. The lysate was added to 5 mL washed Ni-NTA agarose slurry (Qiagen) and loaded into a gravity-flow column (BioRad). After passaging the lysate, the column was washed with 2 column volumes of wash buffer (20 mM HEPES-KOH [pH 7.5], 500 mM NaCl, 40 mM imidazole, 1 mM TCEP, 1% glycerol) and protein was eluted with 10 mL elution buffer (20 mM HEPES-KOH [pH 7.5], 300 mM NaCl, 400 mM imidazole, 1 mM TCEP, 1% glycerol). Fractions containing the most protein were combined and concentrated using a YM10 centrifugal filter (Millipore) before being dialyzed using a 10,000 molecular weight cut-off Slide-a-Lyzer dialysis cassette (Thermo Scientific) into storage buffer (20 mM HEPES-KOH [pH 7.5], 300 mM NaCl, 50 mM KCl, 1 mM TCEP, 5% glycerol, 0.1 mM EDTA). Protein was aliquoted and stored at −80°C. Protein concentration was estimated using the Bradford Assay (Bio-Rad).

To obtain RSP_0847 for antibody production (used in ChIP-seq), after dialysis of RSP_0847-TEV-H_8_, the H_8_ tag was cleaved by incubation with H_6_-TEV protease. The cleaved H_8_ tag and H_6_-TEV protease were removed with a second round of Ni-NTA chromatography. A 5 mL RSP_0847 protein fraction was subjected to size exclusion chromatography in storage buffer through a GE Healthcare HiLoad 16/600 Superdex 75 pg 120mL XK column (Milipore) at a flow rate on 0.5 mL/min controlled by a GE Healthcare ÄKTA Prime Plus FPLC system. Two mL fractions were collected, and sample purity was analyzed by loading ~250 ng of protein on a 12% SDS-PAGE gel. Fractions enriched for RSP_0847 (MW = 28.2 kDa) were combined and concentrated, as above, to ~2 mg/mL. Purified RSP_0847 protein was sent to Covance (Denver, PA) to raise a rabbit polyclonal antibody.

### Phosphotransfer assay

Purified, truncated RSP_1056 (5 μM) was incubated at 30°C for 30 minutes in storage buffer supplemented with 50mM Tris-HCl pH 7.5, 50 mM KCl, 5 mM MgCl_2_, 50 μM ATP, 1 μCi [γ^32^P] ATP (6.16 μCi/μL, [Perkin Elmer]). Purified candidate response regulator proteins were diluted to 5 μM in storage buffer. For each phosphotransfer assay, 5 μL phosphorylated kinase (from the above reaction) and 5 μL response regulator (2.5 μM each protein in storage buffer) were incubated at 30°C. Reactions were stopped with 5 μL 3x NuPAGE LDS (Thermo Fisher) containing 10 mM DTT and stored on ice until samples were loaded, without heating, onto a NuPAGE 4–12% Bis-Tris Protein gel (Invitrogen). Samples were electrophoresed at room temperature for 50 minutes at 150 V. Regions of the gel below the dye front were removed with a razor blade, and the gel was placed between saran wrap and dried for 30 minutes. The dried gel was exposed to a phosphor screen for 3 hours at room temperature before imaging with a Typhoon phosphorimager at 50 μm resolution.

### RNA isolation and sequencing

*Rb*. *sphaeroides* was grown aerobically in 500 mL cultures bubbled with 69% N_2_, 30% O_2_, and 1% CO_2_. 44 mL of cells were harvested when the culture reached an OD_600_ of ~0.5 (mid-log), combined with 6 mL of ice-cold stop solution (95% EtOH and 5% acid phenol:chloroform (5:1, pH 4.5)), and centrifuged at 4°C for 10 minutes at 6,000 x *g*. Cell pellets were resuspended in 2 mL of lysis solution (2% sodium dodecyl sulfate, 16mM EDTA, RNase-free water) and heated at 65°C for 5 minutes. Nucleic acids were extracted using 2 mL of acidic phenol:chloroform heated to 65°C, mixed by inversion, incubated at 65°C for 5 minutes, and centrifuged at room temperature for 7 minutes at 20,000 x *g*. The aqueous phase was removed, extracted twice more, before addition of 2 mL of chloroform, mixed, and centrifuged once more at room temperature for 7 minutes at 20,000 x *g*. The aqueous layer was removed and mixed with 1/10 volume of 3M sodium acetate and equal volume of isopropanol, and incubated at -20°C for >1 hour to precipitate nucleic acids that were collected by centrifugation at 4°C for 30 minutes at 16,000 x *g*. The pellet was washed twice with 1 mL of 75% EtOH (prepared with RNase-free water). Once residual ethanol was removed by evaporation, the pellet was resuspended in 85 μL of RNase-free water. Samples were treated with RNase-free DNase (10 μL 10x DNase buffer, 2 μL DNase, 3 μL RNasin (Promega)) and purified using RNeasy kit (Qiagen).

RNA-seq library preparation and sequencing were performed at the Joint Genome Institute. Sequencing libraries were created using the Illumina TruSeq Stranded Total RNA kit (Illumina) and sequenced on an Illumina NextSeq in 2x151 reads using the manufacturers protocol. The resulting paired-end FASTQ files were split into R1 and R2 files, and R1 files were retained for processing through the same pipeline. Reads were trimmed using Trimmomatic version 0.3 [113] with the default settings except for a HEADCROP of 5, LEADING of 3, TRAILING of 3, SLIDINGWINDOW of 3:30, and MINLEN of 36. After trimming, the reads were aligned to the *Rb*. *sphaeroides* 2.4.1 genome sequence (GenBank accession number GCA_000012905.2) using Bowtie2 version 2.2.2 [114] with default settings except that the number of mismatches was set at 1. Aligned reads were mapped to gene locations using HTSeq version 0.6.0 [115] using default settings except that the “reverse” strandedness argument was used. edgeR version 3.26.8 [116,117] was used to identify significantly differentially expressed genes from pairwise analyses, using Benjamini and Hochberg [118] false discovery rate (FDR) of less than 0.05 as a significance threshold. Raw sequencing reads were normalized using the reads per kilobase per million mapped reads (RPKM). Pathway enrichment was performed using the SmartTable enrichment function at biocyc.org [119] using a P value of ≤ 0.05 as significant.

### Chromatin immunoprecipitation, sequencing, and CenR binding site analysis

*Rb*. *sphaeroides* strains (S2 Table) were grown aerobically in 500 mL culture bubbled (69% N_2_, 30% O_2_, and 1% CO_2_). To each 500 mL culture, 5 mL of 1M sodium phosphate mix (0.85 M Na_2_HPO_4_, 0.15 M NaH_2_PO_4_) and 13 mL of 37% formaldehyde was added. Crosslinking was allowed to continue with continuous bubbling of the culture for 5 minutes. Each culture was placed into an ice bath and 20 mL of ice cold 2.5 M glycine was added and incubated with bubbling for 30 minutes to quench crosslinking. Cells were pelleted in 50 mL portions by centrifugation at 3500 x *g* for 10 minutes at 4°C, washed twice with 100 mL of phosphate buffered saline (PBS), resuspended in 1 mL of PBS, and flash frozen. DNA was isolated in tandem and pooled from technical replicates to improve DNA yields. For DNA isolation, cell pellets were resuspended in 500 μL RIPA buffer (Sigma) supplemented with 50 μL HALT Protease Inhibitor (Thermo). Cells were lysed at 4°C by water bath sonication for 30 cycles at 25% pulsed 25 seconds on and 35 seconds off. To each lysate, 2 μL of micrococcal nuclease (Pierce), 10 μL CaCl_2_ mix (15 mM Tris-HCl pH 8, 1 mM CaCl_2_, 60 mM KCl, 15 mM NaCl, 300 mM sucrose, 0.5 mM DTT), and 10 μL RNase A (0.1 mg/mL final (Fisher)) was added and the mixture was incubated at 4°C for 1 hour. Each nuclease reaction was stopped with 15 mM EDTA and lysate was centrifuged at 16,000 x *g* at 4°C to remove cell debris. DNA fragmentation (major band between 200-400bp) was check by gel electrophoresis on a 1.5% agarose gel. For immunoprecipitation, 1/10 of the volume was taken from each lysate and mixed with an equal volume Protein A sepharose beads (Sigma) to be used as input and negative control. After preclearing the remaining lysate volume with 50 μL Sepharose beads, 5 μL of polyclonal anti-RSP_0847 antibody was added to each sample and incubated at 4°C overnight. Sepharose beads (60 μL) were added to each lysate, the volume was adjusted to ~750 μL with RIPA buffer, and samples were centrifuged for 2 minutes at 1,000 x *g* at 4°C to pellet antibody-protein-DNA complexes bound to the beads. The pelleted beads were washed twice with 1 mL LiCl Wash Solution (100 mM Tris-HCl pH 8, 250 mM LiCl, 2% TritionX-100), twice with 1 mL 600 mM NaCl Wash Buffer (100 mM Tris-HCl pH 8, 600 mM NaCl, 2% TritionX-100), twice with 1 mL 300 mM NaCl Wash Buffer (100 mM Tris-HCl pH 8, 300 mM NaCl, 2% TritionX-100) and twice with TE Buffer (10 mM Tris-HCl pH8, 1 mM EDTA). 150 μL Elution Buffer (50 mM Tris-HCl pH 8, 10 mM EDTA, 1% SDS) was added to the pelleted Sepharose beads and incubated for 1 hour at 65°C. The mixture was centrifuged (1,000 x *g* at 4°C) and the supernatant was removed and incubated overnight at 65°C to reverse-crosslinking. DNA was purified using QIAquick PCR Purification Kit (Qiagen), eluted into a volume of 50 μL and quantified using Qubit Fluorometer 1x dsDNA HS kit (Invitrogen, CA) for sequencing.

ChIP-seq library preparation (starting with at least 20 ng DNA per sample) and sequencing was performed by GeneWiz (South Plainfield, NJ) using an Illumina ChIP-seq Library kit for sequencing with Illumina HiSeq 2x150 bp configuration, single index. The paired-end FASTQ files were trimmed with Trimmomatic version 0.3 [113] with default settings except for LEADING:3, TRAILING:3, SLIDINGWINDOW:3:30, and MINLEN:36. The trimmed paired-end reads were aligned to the *Rb*. *sphaeroides* genome sequence (GenBank accession Chr1: NC_007493.2, Chr2: NC_007494.2) using Bowtie2 version 2.2.2 [114] with default settings except the number of mismatches was set to 1. Picard-tools version 1.98 [120] and Samtools version 1.2 [121] were used to clean, sort, and index the alignment file using default settings. Areas of enrichment of immunoprecipitated over INPUT (peaks) were identified using MOSAiCS version 2.28 using two factor analysis and an analysis type of “IO” [122]. Conversion of BAM to ELAND format for MOSAiCS was performed using Pyicos version 2.0.6 [123] and default settings. WIG files were generated for each sample using QuEST version 2.4 [124] and default settings. WIG files were visualized on MochiView version 1.46 [125].

To identify a potential consensus CenR binding site, sequences 200bp upstream of the translational start site of candidate transcriptional units (S1 Table) were selected and MEME [42,43] was used to search for an enriched motif present in these 59 sequences using the following parameters: -objfun classic -dna -mod zoops -minw 10 -maxw 20 -allw -revcomp. The PWM (S4B Table) generated from this alignment was used to produce the candidate CenR motif (Fig 4A, E-value 2.6e-10). MEME identified this motif in 31 sequences with a p-value < 0.001 for each individual sequence. Orientation and proximity of each candidate sequence to mapped TSS [41] and predicted promoter elements [126] was assessed within the *Rb*. *sphaeroides* 2.4.1 genome.

### Electromobility Shift Assays

DNA fragments (250-300bp) were PCR amplified from genomic DNA and purified via gel excision (Qiagen). H_8_-TEV-CenR was purified as described above and the H_8_ tag was cleaved with H_6_-TEV protease and removed via gravity flow nickel agarose column. 20 μM CenR was phosphorylated in storage buffer supplemented with 25 mM acetyl-phosphate and 20 mM MgCl_2_ and incubated at 37°C for 1.5hrs. EMSA reactions (10 μL total) consisted of a final concentration of 25 mM Tris-HCl pH 7.9, 5 mM EDTA, 10% glycerol, 40 mM potassium glutamate, 20 μg/mL BSA, 1 mM DTT, 200 mM NaCl, and 15 nM DNA substrate. Appropriate dilutions of phosphorylated CenR were made in storage buffer and 1 μL was added to each reaction for a final monomer protein concentration indicated in the figure. Reactions were incubated at 30°C for 30 min, stopped by addition of 5x loading dye (Invitrogen), and loaded onto a 6% nondenaturing polyacrylamide TBE gel, and electrophoresed in 0.5x TBE with 10% glycerol for 2 hours at 100V. Following electrophoresis, the gel was stained with SYBR green (Invitrogen) 1:10,000 in 1x TBE for 30 min in the dark and imaged on a Visi-Blue transilluminator.

### Phylogenetic analysis and identification of CenR binding sites in other *Alphaproteobacteria*

Amino acid sequences of RSP_1056 (CenK) and RSP_0847 (CenR) were used from the *Rb*. *sphaeroides* 2.4.1 genome (Genbank accession numbers ABA80240.1 and ABA80028.1, respectively) and homologs in other α-proteobacteria were identified via BLASTP of non-redundant sequences in Genbank (E-value >1e-70 with a percent identity match >30%) [127,128]. Gene sequences were then aligned in MEGA version 6.06 [129] using MUSCLE [130] and trimmed using Gblocks [131]. Phylogenetic trees were constructed using the Maximum-Likelihood (ML) methods of analysis. The ML analyses for all genes were performed using FastTree 2 [132] with the parameters:–wag,–gamma,–pseudo. Bootstrap values (bs) are reported for the ML trees. Trees were rooted with the *Rhodospirillales* clade to match established α-proteobacteria relationships [59–62]. Trees were colored by order: Grey–*Rhodospirillales*/*Kiloniellales*; Purple–*Sneathiellales*; Red–*Rhodobacterales*; Orange–*Caulobacterales*/*Parvularculales*; Cyan–*Hyphomicobiales*; Blue–*Rhizobiales*.

To investigate possible CenR regulation in other α-proteobacteria, we identified potential homologs of *R*. *sphaeroides* CenR regulated genes in four well studied organisms: *C*. *crescentus* (NA1000, RefSeq accession number GCF_000022005.1), *B*. *abortus* (2308, RefSeq accession number GCF_000054005.1), *A*. *tumefaciens* (BIM B-1315G, RefSeq accession number GCF_014489975.1), and *Rp*. *palustris* (RCB100, RefSeq accession number GCA_000195775.1). Homologs were identified using BLASTn (version 2.9.0, default settings) [127,128]. For each identified homolog from each organism, 300 bp upstream of the translation start site was selected and searched for a match to the *Rb*. *sphaeroides* CenR PWM using PatSer (version 3f, default settings) [133]. For each sequence, the single site with the highest PatSer score was retained. Logos were constructed from the PatSer identified CenR sites using WebLogo [134].

## Supporting information

S1 Fig Other predicted partners of CenK are not phosphorylated *in vitro*. **(A)** Bayesian probabilities for the highest ranked predicted cognate response regulator of RSP_1056. Reciprocal search of this database for cognate kinases of RSP_0847 also identified RSP_1056 as the highest ranked partner. The Bayesian algorithm models amino acid composition of interacting kinase/regulator pairs to predict specific interaction networks for orphaned TCSs across bacterial genomes [27]. **(B)** Phosphotransfer between phosphorylated RSP_1056 and the candidate recombinant response regulators RSP_1083 and RSP_1274 was not detected.
(PDF)

S2 Fig Use of pk18mobsacB-Δ*cenR* supports essentiality of this RR in *Rb*. *sphaeroides*. **(A)** Schematic for the workflow of recombineering in *Rb*. *sphaeroides*. Cloned into the suicide vector, pk18mobsacB, are 1kb DNA fragments corresponding to the sequences immediately 5’ (upstream) and 3’ (downstream) of the desired recombination site. Following successful mating, stable integration of the plasmid into the genome by recombination is selected for first by resistance to kanamycin. The second crossover is initiated by counter selection with sucrose as cells harboring *sacB* do not survive in the presence of sucrose. Thus, a successful double crossover is identified by screening for Kan^S^Suc^R^ colonies. This double crossover can occur at either the original integration site resulting in no change to the chromosome or within the second homologous region resulting in allelic exchange within the target region. **(B)** Deletion of *cenR* is possible in the presence of an ectopic copy of *cenR*. The results of colony PCR screening for the loss of *cenR* (lane 1–3) in strains containing pIND5_spec_-*cenR* (lanes 4–6) relative to WT (lanes 3,6). Colony 1 represents the first possible outcome of the double crossover workflow and colony 2 represents the second possible outcome and successful deletion of *cenR*. **(C)** Deletion of *cenR* in the presence of ectopically expressed *cenR* was successful. Double crossover events were scored (phenotype: Spec^R^Kan^S^Suc^R^), and deletions confirmed by PCR (genotype). **(D)** Plasmid map of pIND5_*spec*_-*cenR* showing the location of primers used to confirm the presence of the plasmid by colony PCR. **(E)** Genomic positions and distances for the primers used in colony PCR to confirm the deletion of *cenR* (WT = 1,837 bp; Δ*cenR* = 1,150 bp).
(PDF)

S3 Fig Analysis of genome-wide transposon insertions within the *cenR* and *cenK* loci. Transposon insertion data determined previously by Burger *et*. *al*. (2017) [33]. The *Rb*. *sphaeroides* Tn5 mutant library was grown aerobically in Sistrom’s minimal medium and split into two replicates (aliquots 1 and 2). Insertion positions and read count data for each genomic region are shown. **(A)** The genomic regions surrounding the nonessential gene *cenK* (*RSP_1056*). **(B)** The genomic region surrounding the essential gene *cenR* (*RSP_0847*).
(PDF)

S4 Fig CenR(D56E) hyperactivity and cell elongation is independent of CenK activity. Bright-field microscopy measurements of the length of exponential phase cells displayed as beeswarm plots [135]. Mean values from each of the three biological replicates (grey circles, yellow triangles, and blue squares, respectively) and the mean value of cell length for each strain is shown (black bar). For each replicate n > 1000 cells were analyzed. Unpaired t-tests were used to compare pooled cell dimension data from the mean values of each biological replicate (n = 3, * *P* value < 0.05). (Mean ± SD) Wild-type: 2.09 ± 0.13 μm; *cenR*(D56E) Δ*cenK*: 2.91 ± 0.05 μm.
(PDF)

S5 Fig CenR binds the promoters of *tolQRAB* and *rpoH1* in vitro. Gel mobility shift assays containing DNA sequences directly upstream of *tolQ* (lanes 1–5), *rpoH1* (lanes 6–10), or *RSP_2157* (lanes 11–15) transcription start sites [41]. To each reaction, 0, 125, 250, 500, or 1000 nM of phosphorylated CenR (CenR~P) was used to shift DNA.
(PDF)

S6 Fig Putative CenR binding and regulation of the division and cell wall gene cluster. Shown are the ChIP-seq data traces of CenR binding upstream of an indicated promoter in TCS “on” strains (*cenR*(D56E) in green, and WT in black) and TCS “low activity” strains (Δ*cenK* in red, and *cenR*(D56A) in orange). ChIP-seq peak heights are represented on the y-axis (fold-enrichment of IP vs input DNA). The chromosomal location is shown on the x-axis with genes represented by arrows pointing in the direction of transcription. TSSs of known promoter(s) are represented as green arrows [41,124]. This inset shows the log_2_ fold change in transcript levels determined from RNA-seq experiments showing change in abundance of the indicated gene in TCS mutant strains relative to WT cells. Non-significant (N.S.) changes in gene expression (FDR > 0.05) are indicated with a grey box. **(A)** The first half of the *dcw* operon showing putative CenR enrichment within of the promoter region of *mraZ*. **(B)** The second half of the *dcw* operon showing CenR enrichment within the promoter regions of *ftsW* / *RSP_2105* and *ddlA*. CenKR TCS activity is predicted to repress the transcription of genes in the *dcw* operon.
(PDF)

S1 Table The expanded CenKR regulon. (PDF)

S2 Table Primers, plasmids, and strains used in this study. (PDF)

S3 Table Genbank accession numbers for protein sequences used to construct CenK/R phylogeny. (PDF)

S4 Table PatSer motif search scores and the probability weight matrix (PWM) of the identified CenR binding motif. (PDF)

S1 Data MOSAiCS ChIP-seq peak dataset. (XLSX)

S2 Data RNA-seq dataset. (XLSX)

S3 Data Raw cell length and width measurements. (XLSX)
