# Supplementary material for: The essential Rhodobacter sphaeroides CenKR two-component system regulates cell division and envelope biosynthesis
Source: PLoS Genet. 2022 Jun 29;18(6):e1010270. doi: 10.1371/journal.pgen.1010270 (PMC9275681; doi:10.1371/journal.pgen.1010270)

A

Kinase	Predicted Regulator	Probability
RSP1056	RSP0847	0.80122
RSP1056	RSP1274	0.14385
RSP1056	RSP1083	0.05493

Regulator	Predicted Kinase	Probability
RSP0847	RSP1056	0.80122
RSP0847	RSP3217	0.00046
RSP0847	RSP0491	0.00013

B

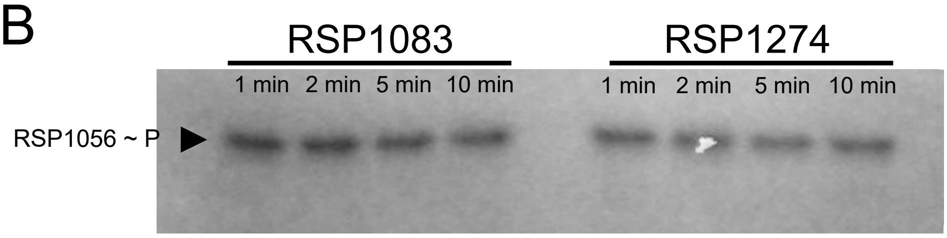

Supplement: S1 Fig — (A) Bayesian probabilities for the highest ranked predicted cognate response regulator of RSP_1056. Reciprocal search of this database for cognate kinases of RSP_0847 also identified RSP_1056 as the highest ranked partner. The Bayesian algorithm models amino acid composition of interacting kinase/regulator pairs to predict specific interaction networks for orphaned TCSs across bacterial genomes [27]. (B) Phosphotransfer between phosphorylated RSP_1056 and the candidate recombinant response regulators RSP_1083 and RSP_1274 was not detected. (PDF) [file pgen.1010270.s001.pdf]
