# Supplementary material for: The essential Rhodobacter sphaeroides CenKR two-component system regulates cell division and envelope biosynthesis
Source: PLoS Genet. 2022 Jun 29;18(6):e1010270. doi: 10.1371/journal.pgen.1010270 (PMC9275681; doi:10.1371/journal.pgen.1010270)

A

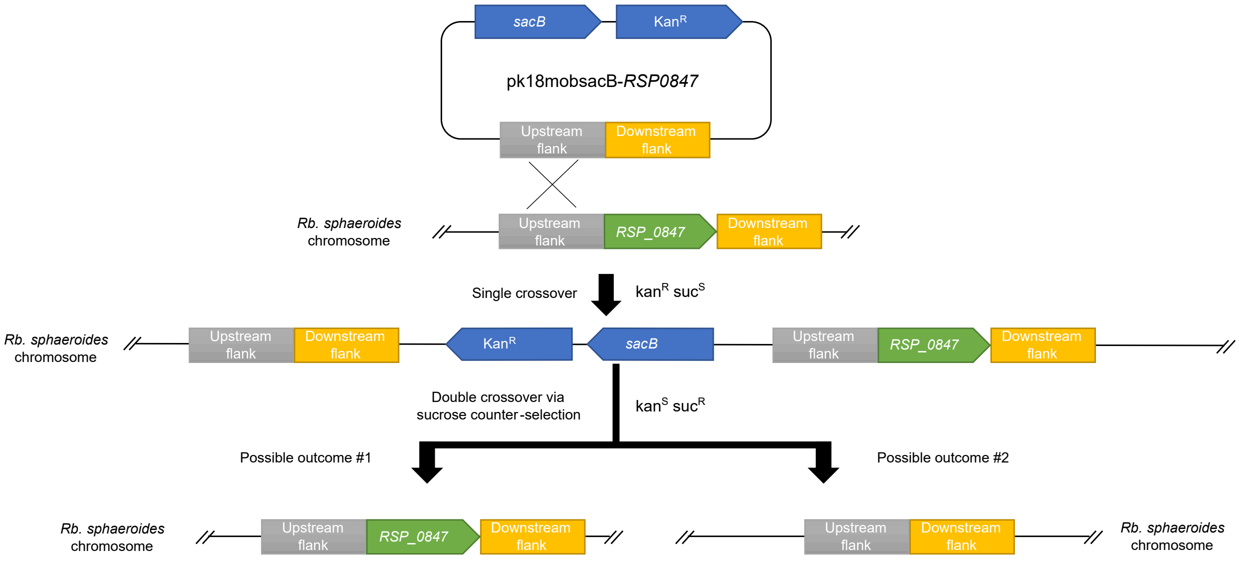

B

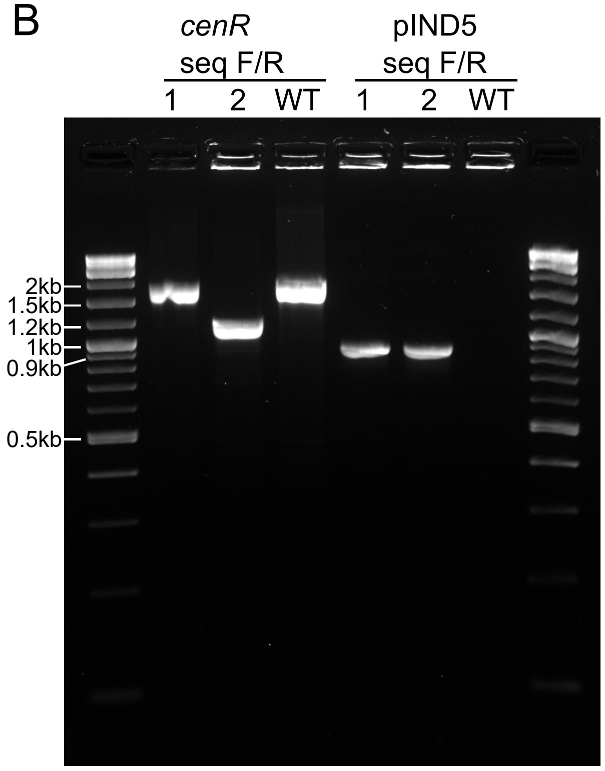

C

		Genotype		Phenotype
**Strain Construction**	**Total Colonies Screened**	**Δ**	**WT**	***sacB* inactivation**
Deletion of *cenR* in the presence of pIND5$_{spec}$-*cenR*	50	16	19	15

D

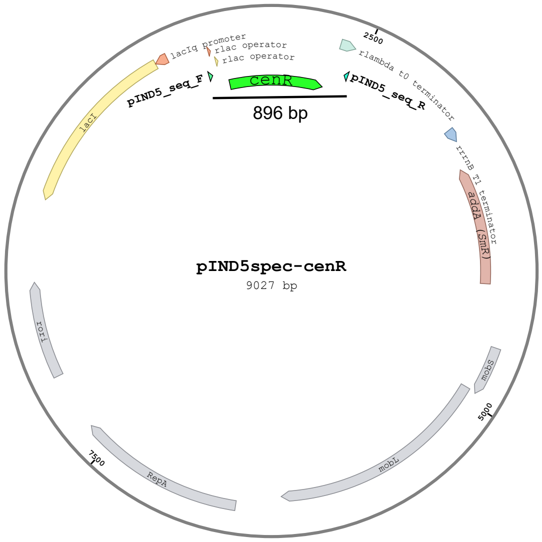

E

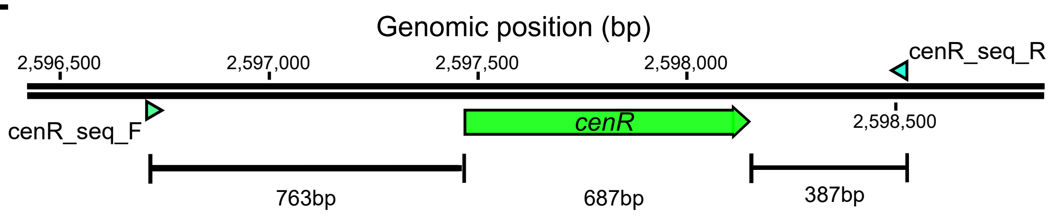

Supplement: S2 Fig — (A) Schematic for the workflow of recombineering in Rb. sphaeroides. Cloned into the suicide vector, pk18mobsacB, are 1kb DNA fragments corresponding to the sequences immediately 5’ (upstream) and 3’ (downstream) of the desired recombination site. Following successful mating, stable integration of the plasmid into the genome by recombination is selected for first by resistance to kanamycin. The second crossover is initiated by counter selection with sucrose as cells harboring sacB do not survive in the presence of sucrose. Thus, a successful double crossover is identified by screening for KanSSucR colonies. This double crossover can occur at either the original integration site resulting in no change to the chromosome or within the second homologous region resulting in allelic exchange within the target region. (B) Deletion of cenR is possible in the presence of an ectopic copy of cenR. The results of colony PCR screening for the loss of cenR (lane 1–3) in strains containing pIND5spec-cenR (lanes 4–6) relative to WT (lanes 3,6). Colony 1 represents the first possible outcome of the double crossover workflow and colony 2 represents the second possible outcome and successful deletion of cenR. (C) Deletion of cenR in the presence of ectopically expressed cenR was successful. Double crossover events were scored (phenotype: SpecRKanSSucR), and deletions confirmed by PCR (genotype). (D) Plasmid map of pIND5spec-cenR showing the location of primers used to confirm the presence of the plasmid by colony PCR. (E) Genomic positions and distances for the primers used in colony PCR to confirm the deletion of cenR (WT = 1,837 bp; ΔcenR = 1,150 bp). (PDF) [file pgen.1010270.s002.pdf]
