# Supplementary figures and images for: The essential Rhodobacter sphaeroides CenKR two-component system regulates cell division and envelope biosynthesis
Source: PLoS Genet. 2022 Jun 29;18(6):e1010270. doi: 10.1371/journal.pgen.1010270 (PMC9275681; doi:10.1371/journal.pgen.1010270)

**A**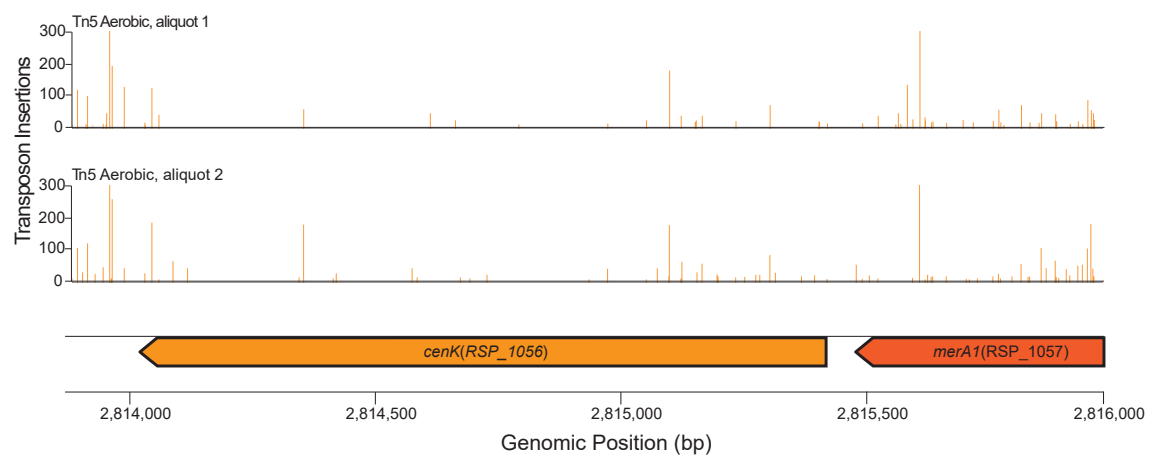**B**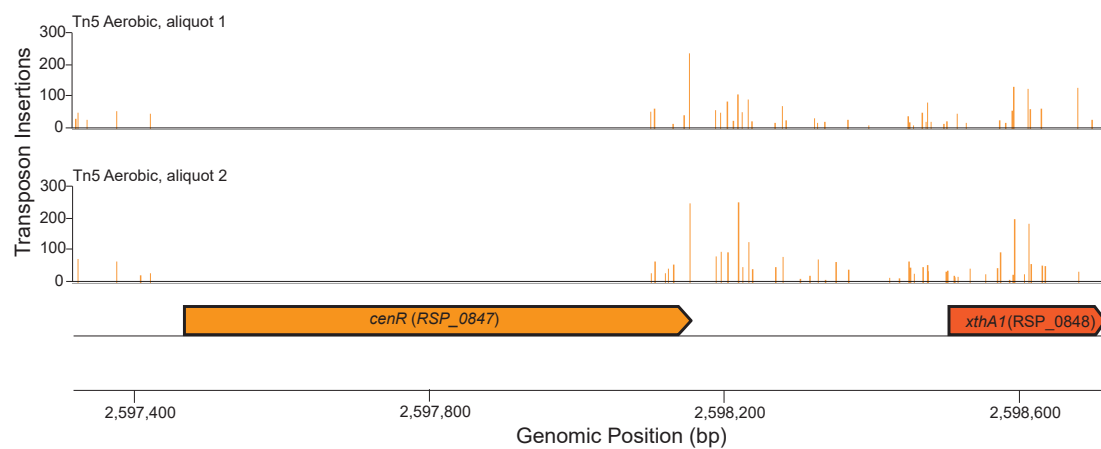

Supplement: S3 Fig — Transposon insertion data determined previously by Burger et. al. (2017) [33]. The Rb. sphaeroides Tn5 mutant library was grown aerobically in Sistrom’s minimal medium and split into two replicates (aliquots 1 and 2). Insertion positions and read count data for each genomic region are shown. (A) The genomic regions surrounding the nonessential gene cenK (RSP_1056). (B) The genomic region surrounding the essential gene cenR (RSP_0847). (PDF) [file pgen.1010270.s003.pdf]

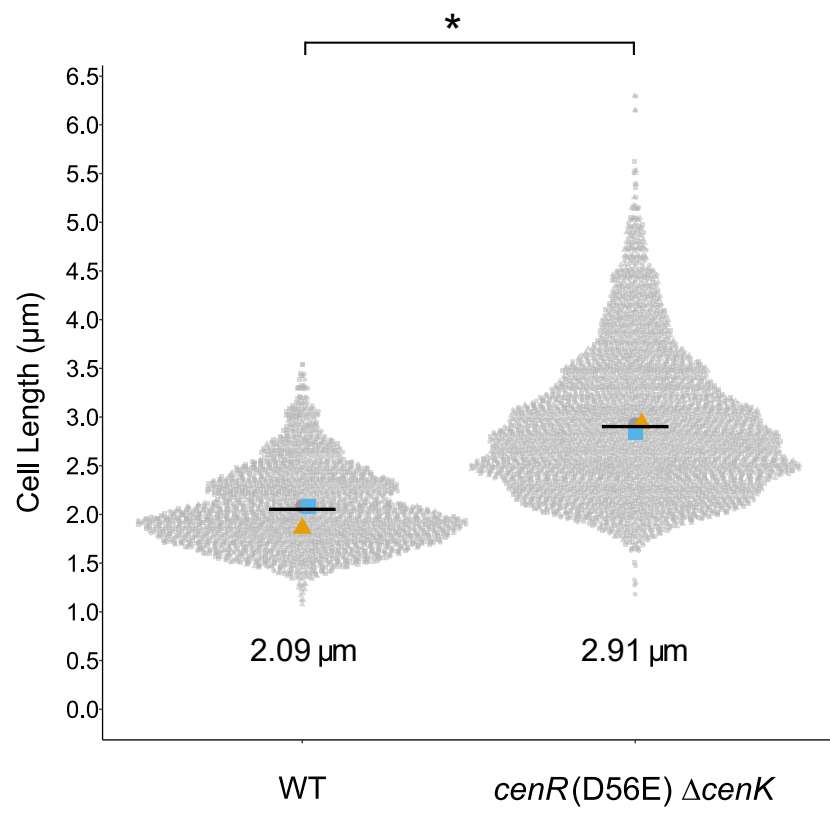

Supplement: S4 Fig — Bright-field microscopy measurements of the length of exponential phase cells displayed as beeswarm plots [135]. Mean values from each of the three biological replicates (grey circles, yellow triangles, and blue squares, respectively) and the mean value of cell length for each strain is shown (black bar). For each replicate n > 1000 cells were analyzed. Unpaired t-tests were used to compare pooled cell dimension data from the mean values of each biological replicate (n = 3, * P value < 0.05). (Mean ± SD) Wild-type: 2.09 ± 0.13 μm; cenR(D56E) ΔcenK: 2.91 ± 0.05 μm. (PDF) [file pgen.1010270.s004.pdf]

*tolQRAB* *rpoH1* *RSP\_2157*

CenK~P: 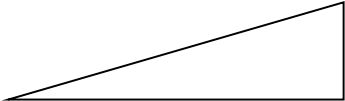 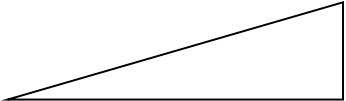 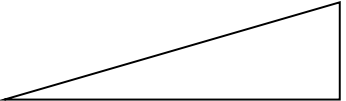

Lane: 1 2 3 4 5 6 7 8 9 10 11 12 13 14 15

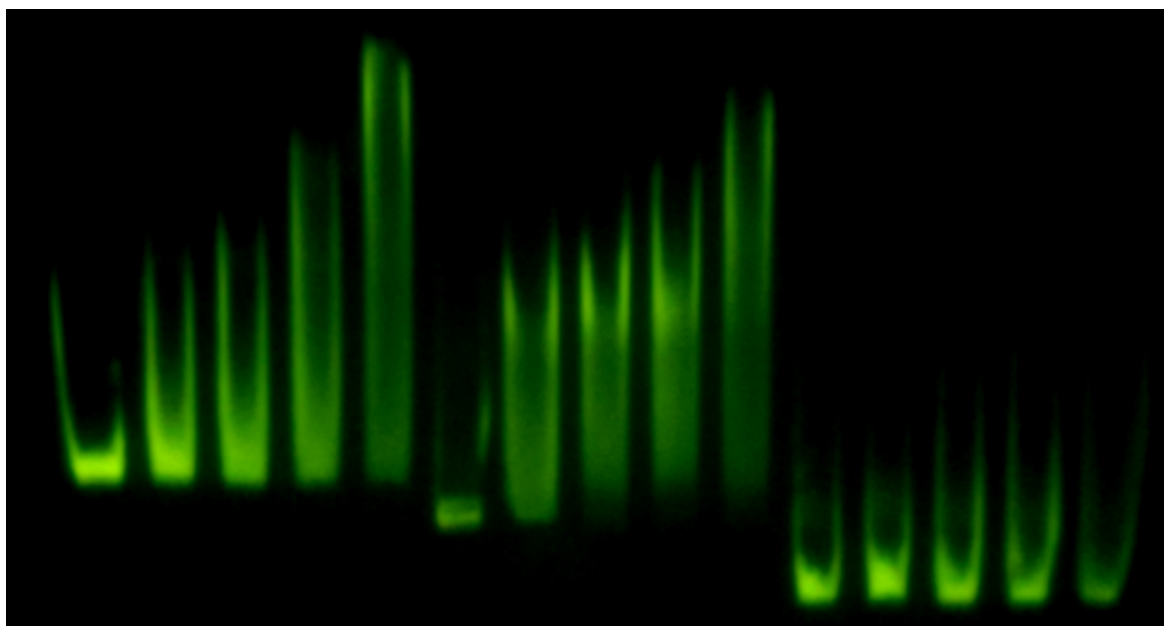

Supplement: S5 Fig — Gel mobility shift assays containing DNA sequences directly upstream of tolQ (lanes 1–5), rpoH1 (lanes 6–10), or RSP_2157 (lanes 11–15) transcription start sites [41]. To each reaction, 0, 125, 250, 500, or 1000 nM of phosphorylated CenR (CenR~P) was used to shift DNA. (PDF) [file pgen.1010270.s005.pdf]

A

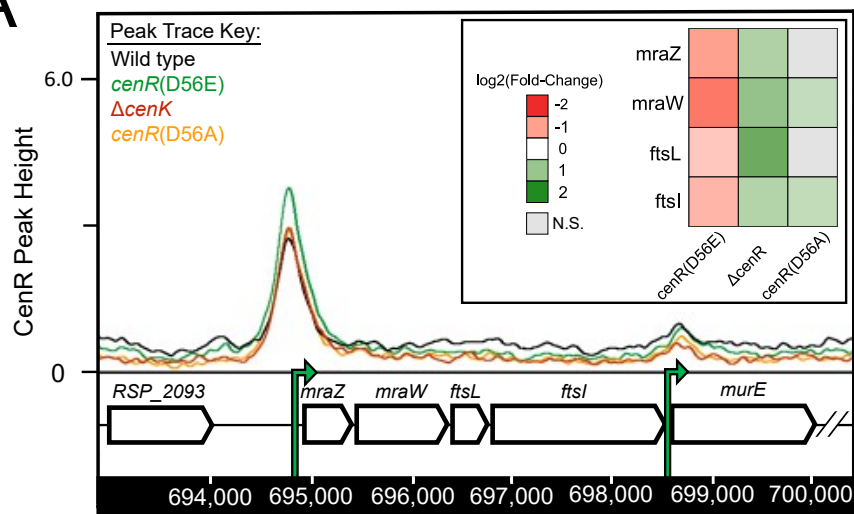

B

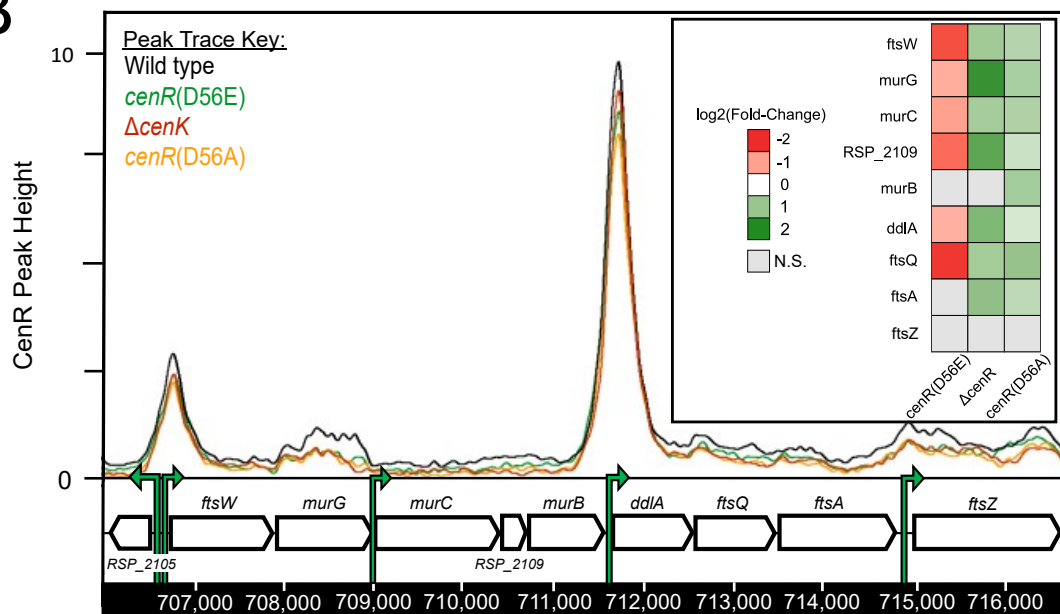

Supplement: S6 Fig — Shown are the ChIP-seq data traces of CenR binding upstream of an indicated promoter in TCS “on” strains (cenR(D56E) in green, and WT in black) and TCS “low activity” strains (ΔcenK in red, and cenR(D56A) in orange). ChIP-seq peak heights are represented on the y-axis (fold-enrichment of IP vs input DNA). The chromosomal location is shown on the x-axis with genes represented by arrows pointing in the direction of transcription. TSSs of known promoter(s) are represented as green arrows [41,124]. This inset shows the log2 fold change in transcript levels determined from RNA-seq experiments showing change in abundance of the indicated gene in TCS mutant strains relative to WT cells. Non-significant (N.S.) changes in gene expression (FDR > 0.05) are indicated with a grey box. (A) The first half of the dcw operon showing putative CenR enrichment within of the promoter region of mraZ. (B) The second half of the dcw operon showing CenR enrichment within the promoter regions of ftsW / RSP_2105 and ddlA. CenKR TCS activity is predicted to repress the transcription of genes in the dcw operon. (PDF) [file pgen.1010270.s006.pdf]
