# Supplementary material for: The essential Rhodobacter sphaeroides CenKR two-component system regulates cell division and envelope biosynthesis
Source: PLoS Genet. 2022 Jun 29;18(6):e1010270. doi: 10.1371/journal.pgen.1010270 (PMC9275681; doi:10.1371/journal.pgen.1010270)
Supplement: S1 Table — (PDF) [file pgen.1010270.s007.pdf]

CENTER OF PEAK (bp)[a]	ChIP-seq FOLD ENRICHMENT (IP/INPUT)				DOWNSTREAM GENE			Transmembrane Domain(s)[b]	Signal Peptide[c]	Sequence Logo Predicted DNA-binding motif: TGA-N8-TGA	TRANSCRIPT FOLD CHANGE (log2)[d]						REGULATORY ROLE[e]	Essential[f]
	D56E	WT	ΔcenK	D56A	ID	GENE NAME	ANNOTATION				cenR(D56E)	FDR	ΔcenK	FDR	cenR(D56A)	FDR		
**Cell envelope, transport, and extracytoplasmic targeted proteins**																		
2597500	1.4	--	--	--	RSP_0847	*RSP_0847*	two component transcriptional regulator winged helix family	N	N	ACACGAGCGCGTGAG	▲ 0.49	9.1E-07	▼ -5.16	5.9E-07	▼ -4.34	1.1E-06	+	Y
2413901	4.5	3.2	1.6	1.6	RSP_0672	*tolQ*	Cell division and transport-associated protein TolQ	Y	N	TGACGCAGATGTGTT	▲ 2.51	2.3E-03	▼ -0.58	6.7E-03	n.s.	n.s.	+	Y
392575	3.8	1.7	--	--	RSP_1807	*RSP_1807*	putative secreted protein	N	Y	TCGCAAAACCTTGAT	▲ 2.72	4.1E-06	▼ -2.04	1.4E-04	▼ -1.97	8.0E-05	+	N
426084	2.8	2.3	--	--	RSP_1834	*RSP_1834*	Beta-lactamase superfamily	N	Y	TGACGCAGCTGTGAA	▲ 2.47	4.4E-06	▼ -5.56	1.7E-05	▼ -5.43	2.8E-07	+	N
2970115	2.9	2.2	--	--	RSP_1199	*RSP_1199*	L,D-transpeptidases/carboxypeptidases	N	Y	TGACGCGGGCGTGAA	▲ 2.47	4.4E-05	▼ -1.98	9.9E-05	▼ -1.39	5.1E-04	+	N
2970115*	2.9*	2.2*	--	--	RSP_1200	*RSP_1200*	uncharacterized protein with SCP/PR1 domains	Y	Y	TGAGTTGCATTTGAA	▲ 0.52	1.6E-05	▼ -0.52	3.7E-03	n.s.	n.s.	+	N
478200	2.0	1.6	--	--	RSP_1880	*RSP_1880*	peptidoglycan-binding domain-containing protein	N	Y	TCACATGGCCGTGTT	▼ -1.80	4.6E-06	▲ 1.24	5.3E-03	n.s.	n.s.	-	N
2933000	1.8	1.4	--	--	RSP_1168	*surA*	PpiC-type peptidyl-prolyl cis-trans isomerase	N	Y	TGACATGATCGTGTG	▲ 3.40	2.8E-06	▼ -1.48	9.1E-04	▼ -0.74	1.1E-02	+	N
2933000*	1.8*	1.4*	--	--	RSP_1169	*secA*	protein translocase subunit secA	N	N	TCGCGCGACGTTGAC	▲ 1.80	3.0E-06	▼ -0.94	1.7E-03	n.s.	n.s.	+	Y
435330	2.0	--	--	--	RSP_1844	*RSP_1844*	putative periplasmic protein	N	Y	TGGCGCAGGCGTGAC	▲ 1.25	2.2E-05	n.s.	n.s.	n.s.	n.s.	+	N
1368770	2.0	--	--	--	RSP_2718	*RSP_2718*	putative outer membrane protein	Y	Y	TCACCTCCTCGTGAG	▲ 3.09	8.2E-04	▼ -4.76	5.9E-07	▼ -6.49	2.9E-07	+	N
2641460	1.8	--	--	--	RSP_0890	*mlaC*	Intermembrane phospholipid transport system binding protein	N	Y	TCAATACTTGGTGAT	▲ 2.47	1.7E-03	▼ -0.74	2.2E-03	▼ -1.14	6.9E-04	+	N
2473818	3.2	1.9	--	--	RSP_0730	*RSP_0730*	TIGR02302 family protein	Y	N	TCACTTCGCCGTCAT	▲ 2.23	2.0E-04	▼ -1.63	3.7E-04	▼ -0.63	1.5E-02	+	N
2450500	2.0	1.4	--	--	RSP_0704	*RSP_0704*	ABC peptide transporter substrate binding protein	Y	Y	TCACGCAATCGCAAC	▲ 1.77	2.1E-05	▼ -0.62	4.0E-03	n.s.	n.s.	+	N
458700	1.6	1.7	--	--	RSP_1860	*RSP_1860*	cell wall hydrolyses involved in spore germination	N	Y	TGGCAAAAATGTCAC	▲ 1.42	2.1E-03	▼ -0.61	1.1E-02	▼ -1.25	6.6E-04	+	N
1458970	1.6	1.6	--	1.6	RSP_2847	*RSP_2847*	putative lipoprotein (predicted lipid binding domain)	Y	Y	TCTTCGTCCCCTGAC	▼ -1.20	1.1E-03	▲ 0.97	2.7E-02	n.s.	n.s.	-	N
2665900	1.5	--	--	--	RSP_0915	*RSP_0915*	putative periplasmic protein	N	Y	TGACAAACGTGTTAG	▲ 1.43	5.7E-04	n.s.	n.s.	n.s.	n.s.	+	N
606400	1.6	--	--	--	RSP_2006	*RSP_2006*	uncharacterized protein involved in outer membrane biogenesis	Y	N	TCACGCAATCCTGAC	▲ 0.67	7.9E-04	n.s.	n.s.	n.s.	n.s.	+	N
930800 (chr2)	1.7	1.6	--	--	RSP_1413	*RSP_1413*	TRAP-T family transporter periplasmic binding component	Y	Y	TCACAAATGCATTAC	▼ -1.34	1.2E-02	▲ 0.89	5.8E-02	n.s.	n.s.	-	N
1207306	2.7	1.8	--	1.5	RSP_2561	*exoP*	putative succinoglycan biosynthesis transport protein ExoP	Y	N	TCACGATCCTCTCAG	n.s.	n.s.	n.s.	n.s.	▼ -0.60	4.2E-02	+	N
278100	1.7	--	--	--	RSP_1024	*RSP_1024*	Putative MoxR family protein	Y	N	TCACGATCATTTGTG	▲ 0.85	9.1E-05	▼ -0.56	6.1E-03	▼ -0.84	3.8E-03	+	N
2758300	2.8	1.5	--	--	RSP_1000	*dsbC*	Disulphide bond corrector protein DsbC	N	Y	TGGCGCAGTTGTGAC	▲ 2.48	5.9E-06	▼ -1.64	1.9E-03	▼ -2.07	8.8E-05	+	N
2758300*	2.8*	1.5*	--	--	RSP_0999	*RSP_0999*	putative transcriptional regulator	N	N	TCGCGCGACTGTGGC	▲ 1.37	4.5E-05	n.s.	n.s.	n.s.	n.s.	+	N
1273100	1.6	--	--	--	RSP_2633	*ccmF*	Cytochrome c maturation protein CcmF	Y	N	TGACGGGAAGGCGAC	▲ 1.06	3.2E-02	▼ -0.21	3.0E-02	n.s.	n.s.	+	Y
1330900	2.2	--	--	--	RSP_2685	*cycH*	Putative cytochrome c-type biogenesis protein cycH	Y	N	TGACGGGATATTGAC	▲ 0.79	1.1E-04	n.s.	n.s.	▼ -0.89	1.6E-02	+	Y
1330900*	2.2*	--	--	--	RSP_2686	*soxB*	putative sarcosine oxidase beta subunit	N	N	TCGCAACTCGGTGAT	▲ 3.76	6.0E-05	▼ -1.21	5.5E-04	n.s.	n.s.	+	N
51549	1.9	1.5	--	--	RSP_1465	*RSP_1465*	putative aminoglycoside phosphotransferase	N	N	TCACGCAATCTTTAT	▲ 0.78	4.5E-05	▼ -1.14	1.2E-03	▼ -1.42	1.6E-03	+	N
106900 (chr2)	1.6	--	--	--	RSP_3067	*RSP_3067*	hypothetical protein	Y	N	TCGCAGGATCTTGAT	▲ 4.51	1.9E-06	n.s.	n.s.	▼ -1.27	8.0E-03	+	N
84880	2.5	1.6	--	--	RSP_1496	*RSP_1496*	Lysozome-like putative lipoprotein	N	Y	TCGCGTTTTTCTGAA	▲ 1.37	1.3E-03	n.s.	n.s.	n.s.	n.s.	+	N
694770	3.6	2.6	3.0	3.1	RSP_2095	*mraZ*	Putative MraZ protein	N	N		▼ -1.00	8.0E-02	n.s.	n.s.	n.s.	n.s.	-	N
706700	2.0	2.3	2.5	2.5	RSP_2106	*ftsW*	cell division protein FtsW	Y	N		▼ -2.25	1.2E-03	▲ 1.18	1.3E-02	▲ 0.92	1.6E-03	-	Y
711700	7.2	9.9	7.5	5.7	RSP_2111	*ddlA*	D-alanine--D-alanine ligase	N	N		▼ -1.06	2.4E-03	▲ 1.57	3.6E-03	▲ 0.49	1.9E-02	-	Y
2889120	2.6	1.8	--	--	RSP_1125	*mltB*	Membrane bound lytic murein transglycosylase B	N	Y		▲ 1.98	6.5E-06	n.s.	n.s.	n.s.	n.s.	+	N
2430800	1.6	1.7	2.1	1.8	RSP_0688	*dacC*	D-alanyl-D-alanine carboxypeptidase	Y	Y		▼ -0.96	3.2E-03	▲ 1.09	1.5E-02	▲ 0.49	2.0E-02	-	Y
1622411	2.3	2.4	2.7	2.7	RSP_2942	*RSP_2942*	sporulation and cell division-related protein	Y	N		▼ -0.53	1.4E-03	▲ 1.55	8.0E-03	▲ 0.83	3.2E-03	-	Y
239600 (chr2)	1.5	1.8	1.7	1.7	RSP_3186	*RSP_3186*	putative transmembrane protein	Y	N		▼ -1.42	2.3E-02	n.s.	n.s.	n.s.	n.s.	-	N
1360340	1.7	1.8	1.6	1.6	RSP_2711	*RSP_2711*	Beta-barrel assembly machine subunit BamA	Y	Y		▼ -0.42	3.7E-02	n.s.	n.s.	n.s.	n.s.	-	Y
1184160	2.3	1.8	1.7	1.7	RSP_2540	*tatA*	twin-arginine translocation system protein TatA	Y	N		▲ 1.96	4.3E-03	▼ -0.43	1.1E-02	n.s.	n.s.	+	N
1193700	1.6	--	--	--	RSP_2549	*exoQ*	putative polysaccharide polymerase	Y	N		▲ 1.04	9.9E-03	n.s.	n.s.	n.s.	n.s.	+	N
1190291	2.5	3.1	2.9	2.9	RSP_2547	*exoY*	exopolysaccharide production protein exoY	Y	N		▲ 1.26	5.3E-03	n.s.	n.s.	▼ -1.04	2.8E-03	+	N
1426500	2.1	3.2	2.5	2.2	RSP_2778	*RSP_2778*	Invasion protein B involved in pathogenesis	N	Y		▲ 0.55	8.3E-03	▼ -0.29	1.3E-02	n.s.	n.s.	+	N
1670913	2.6	2.4	--	--	RSP_2980	*aspAT*	Putative aspartate aminotransferase A	N	N		▼ -1.44	7.1E-03	n.s.	n.s.	n.s.	n.s.	-	N
1670913*	2.6*	2.4*	--	--	RSP_2981	*RSP_2981*	Putative protein-disulfide isomerase	N	Y		▲ 2.25	3.0E-06	▼ -1.42	6.0E-04	▼ -0.60	5.0E-02	+	N
889240	2.5	2.1	--	--	RSP_2271	*RSP_2271*	putative lipoprotein	N	Y		▲ 1.64	2.8E-02	▼ -0.84	7.2E-03	n.s.	n.s.	+	N
2682500	1.8	--	--	--	RSP_0932	*ctpA*	Peptidase family S41	Y	Y		▲ 1.54	7.0E-05	▼ -0.35	1.7E-02	n.s.	n.s.	+	N
2856500	1.9	1.5	--	--	RSP_1097	*pqqL*	putative zinc protease	N	Y		▲ 3.36	1.8E-05	▼ -1.15	3.5E-03	▼ -1.04	1.0E-03	+	N
740875	3.0	3.2	2.8	2.6	RSP_2140	*RSP_2140*	Protein of unknown function (DUF1194)	N	Y		▼ -1.28	1.6E-05	▲ 1.67	4.3E-02	n.s.	n.s.	-	N
1472000	2.1	2.2	1.9	1.8	RSP_2839	*ntrY*	NtrY Signal transduction histidine kinase	Y	N		▼ -1.46	6.4E-03	▲ 0.78	5.4E-02	▲ 0.58	3.8E-02	-	N
742800	1.9	1.6	1.8	1.6	RSP_2141	*RSP_2141*	ABC polyamine transporter periplasmic substrate-binding protein	N	Y		▲ 1.11	9.4E-05	▼ -0.46	1.2E-02	n.s.	n.s.	+	N
1371600	1.5	1.7	1.6	1.5	RSP_2721	*RSP_2721*	hypothetical protein	N	Y		▼ -0.56	2.0E-02	▲ 1.10	1.3E-02	▲ 0.87	1.3E-03	-	N
**Cytoplasmic proteins**																		
1039245	4.1	2.5	--	--	RSP_2410	*rpoH1*	RNA polymerase sigma 32 subunit - RpoH	N	N	TTACATTCGCGTGAT	▲ 3.20	3.2E-04	▼ -3.38	1.4E-06	▼ -2.30	4.6E-06	+	N
2296600	1.6	--	--	--	RSP_0559	*msrA*	Peptide methionine sulfoxide reductase	N	N	TGGCGCGAGCGTGAT	▲ 3.11	8.92-06	▼ -1.78	1.6E-04	▼ -1.41	4.1E-04	+	N
165500	1.5	--	--	--	RSP_1572	*RSP_1572*	Heat shock protein Hsp20 family	N	N		▲ 0.46	1.4E-02	▼ -0.39	6.4E-03	▼ -0.78	1.2E-02	+	N
1511300	1.8	1.6	--	--	RSP_2806	*Lon*	ATP-dependent proteinase	N	N		▲ 1.08	1.8E-03	▼ -0.54	7.1E-03	n.s.	n.s.	+	N
67000 (chr2)	1.9	2.3	2.1	2.2	RSP_4110	*wbyL*	glycosyl transferase	N	N		▲ 0.86	5.1E-02	n.s.	n.s.	n.s.	n.s.	+	N
2526668	2.7	3.3	3.0	2.7	RSP_0782	*RSP_0782*	Ppx/GppA phosphatase	N	N		▼ -1.52	1.2E-02	▲ 0.89	2.6E-02	n.s.	n.s.	-	N
62100 (chr2)	1.8	2.1	1.8	1.7	RSP_3989	*RSP_3989*	NAD-dependent epimerase/dehydratase	N	N		▼ -2.32	8.8E-04	▲ 1.30	7.1E-03	n.s.	n.s.	-	N
1418670	3.4	4.1	3.7	3.3	RSP_2771	*RSP_2771*	putative Exopolyphosphatase	N	N		▼ -1.01	4.5E-02	▲ 1.26	4.9E-03	n.s.	n.s.	-	N
2931800	1.6	--	--	--	RSP_1167	*argJ*	glutamate N-acetyltransferase	N	N		▲ 1.92	2.3E-04	▼ -1.02	1.7E-03	▼ -0.47	4.3E-02	+	N

a Chromosomal position of ChIP-seq peak (chr2 - chromosome 2)
b Transmembrane domain predicted using TMHMM 2.0 [132]
c Signaling transit peptides identified using SignalP 5.0 [133], scores ≥ 0.6 were interpreted to have predicted transit peptides
d log_2(Fold-Change) are reported relative to WT (i.e. mutant/WT)
e Regulatory role of CenR based on changes in gene expresion between TCS mutants relative to WT cells. (+) positively regulated by CenR, (-) negatively regulated by CenK, (NA) Not assigned from data
f Essentiality determined previously by Tn-seq analysis [33]
-- No significant ChIP enrichment
* Peak located between differentially expressed genes
n.s. Not significant, RNA FDR p-value > 0.05 cutoff
