# Supplementary material for: The essential Rhodobacter sphaeroides CenKR two-component system regulates cell division and envelope biosynthesis
Source: PLoS Genet. 2022 Jun 29;18(6):e1010270. doi: 10.1371/journal.pgen.1010270 (PMC9275681; doi:10.1371/journal.pgen.1010270)
Supplement: S2 Table — (PDF) [file pgen.1010270.s008.pdf]

Table S2. Primers, plasmids, and strains lists.

A. Primers

Name	Sequence	Purpose
BDL3-3	TAGAAAGCCAGTCCGCAGAAAC	PCR Linearization of pk18mobsacB
BDL3-4	CTGTCGTGCCAGCTGCATTAATGAATCG	
BDL77-2	TTAATGCAGCTGGCACGACAGCCTGACGAACGAGACG	PCR upstream flank for RSP1056 deletion vector
BDL78-2	CCGACAGATCAGATCTTGTCCTTCCGAC	
BDL79-2	GACAAGATCTGATCTGTCGGCCCCTC	PCR downstream flank for RSP1056 deletion vector
BDL80-2	CTGCGGACTGGCTTTCTACGATCCGCGCGGGCTCG	
BDL1-3	ATCCCCTGATTCTGTGGATAACCGTATTACCG	PCR amplification of pk18mobsacB cloning site
BDL2-3	CCCAGTCTAGCTATCGCCATGTAAGCC	
BDL74-1	GCTCAACGAACTGTCGAAAC	Sequencing primers of RSP1056 deletion
BDL75-1	CCGGTTCGCTTCTGATGAT	
BDL77-3	TAATGCAGCTGGCACGACAGATTGAGATAGAGGAGCACG	PCR amplification of upstream flank for RSP0847 deletion vector
BDL78-3	CTTAATTTGATCCGGTCACTCCTTCGTTCGTG	
BDL79-3	AGGAGTGACCGGATCAAATTAAGGATCACACTC	PCR amplification of downstream flank for RSP0847 deletion vector
BDL80-3	TTCTGCGGACTGGCTTTCTAGTAGGACCACCAGGAATAGAG	
BDL43-3 (cenR_seq_F)	TCGATGGCGTAATGTTCTTCGC	Sequencing primers of RSP0847 deletion
BDL4-4 (cenR_seq_R)	GACCGAGTTGATGTTCCAGGTG	
BDL77-3	TAATGCAGCTGGCACGACAGATTGAGATAGAGGAGCACG	PCR RSP0847D56A upstream flank for pk18mobsacB
BDL37-4	AGACCCACGGCGAGGATCACGAGGTCGTAGAG	
BDL36-4	TGATCCTCGCCGTGGGTCTGCCGGACAC	PCR RSP0847D56A downstream flank for pk18mobsacB
BDL11-4	TTCTGCGGACTGGCTTTCTACCGTTGTAGCTCTTCTGTC	
BDL77-3	TAATGCAGCTGGCACGACAGATTGAGATAGAGGAGCACG	PCR RSP0847D56E upstream flank for pk18mobsacB
BDL9-4	AGACCCACCTCGAGGATCACGAGGTCGTAGAG	
BDL10-4	TGATCCTCGAGGTGGGTCTGCCGGACAC	PCR RSP0847D56E downstream flank for pk18mobsacB
BDL11-4	TTCTGCGGACTGGCTTTCTACCGTTGTAGCTCTTCTGTC	
BDL12-4	CTTTCCGCTTCTTCCTAGCCAG	Sequencing primers for RSP0847 variants

BDL13-4	GAAGGGCTTCGTCACATAGTCG	
BDL1-5	ATTTTCAGAGCGCGATCGCAGGAATGACCGCCTATGCCGAAGCG	RSP1056-cyto (truncated at M195) insertion into pVP302K with N-terminal H8-TEV tag
BDL2-5	TTGTTATTTTCGGCTTTCTGTCAGACGAGGGCGCTGAGACC	
BDL3-5	GAGGAGAAATTAACCATGGCTTCCCTGAAGAAGATCC	RSP0847 (WT, D56A) insertion into pVP302K with C-terminal TEV-H8
BDL4-5	TTCGCTTCCTGCGATCGCCGCAACAAGCCTGTAGCC	
BDL7-5	AAAGAGGAGAAATTAACCATGACCTCCGACACGACGC	RSP1274 insertion into pVP302K with C-terminal TEV-H8
BDL8-5	TTTTCGCTTCCTGCGATCGCGGCGCTGAGCGTTTCGGTCG	
BDL9-5	AAGAGGAGAAATTAACCATGGTCCCGTGCATTCTTGCGCTC	RSP1083 insertion into pVP302K with C-terminal TEV-H8
BDL10-5	TTTTCGCTTCCTGCGATCGCGCCGGCGAAGGTGTAGCC	
BDL11-5	ACATTTCCCCGAAAAGTGCCAC	pVP302K sequencing primers
BDL12-5	CTTTGTTAGCAGCCGGATCAGC	
BDL41-1	CATATGTAATTTCTCCTC	ATW linearization of $pIND5_{spec}$
BDL42-1	GGATCCAGATCTCATCACCATC	
BDL75-3	AAGAGGAGAAATTACATATGGCTTCCCTGAAGAAGATCC	Amplification of the coding region of *cenR* for assembly into $pIND5_{spec}$
BDL76-3	GATGAGATCTGGATCCTCCTCACGCAACAAGCCTGTAGCCG	
BDL33-1	CTTGTGAGCGGATAACAATGATAC	Sequencing primers for $pIND5_{spec}$
BDL34-1	CAACCGAGCGTTCTGAACAAATCC	
BDL16-8	CGAACTTCACGGTGAGCGTTG	Amplification of tolQ promoter region for EMSA
BDL17-8	GCATCCTCCTCGATCAGGAG	
BDL18-8	CAAGCTCTCGCCGAAGG	Amplification of rpoH1 promoter region for EMSA
BDL19-8	TGTAAGTGCTCATTCGTTGAC	
BDL20-8	CCGTCCCTTCGACCCGCCTC	Amplification of RSP2175 promoter region for EMSA
BDL21-8	GCAGGTGGCGGAACCGGGAG	
BDL22-8	CAGAAAGCCGAAAATAACAAAG	Linearization of pVP302K with N-terminal H_8 tag
BDL23-8	TCCTGCGATCGCGCTCTGAAAATAC	
BDL24-8	GTATTTTCAGAGCGCGATCGCAGGAATGGCTTCCCTGAAGAAGATCC	Amplification of 0847D56E gene for cloning into pVP302K-N
BDL25-8	AACTTTGTTATTTTCGGCTTTCTGTCACGCAACAAGCCTGTAGC	
BDL26-8	ATCCTCGAGGTGGGTCTGCCGGACACC	ATW linearization primers to make pVP302K-N-0847 from pVP302K-N-0847D56E
BDL27-8	AGACCCACCTCGAGGATCACGAGGTCG	

B. Plasmids

Name	Description	Source
pk18mobsacB	Broad host range mobilizable vector; Km^R oriT(RP4) mobT *sacB lacZα*	[1]
pk18mobsacB-RSP1056	*R. sphaeroides* genomic region flanking *RSP1056* Gibson assembled into pk18; Km^R; for deletion of *RSP1056*	This work
pk18mobsacB-RSP0847	*R. sphaeroides* genomic region flanking *RSP0847* Gibson assembled into pk18; Km^R; for deletion of *RSP0847*	This work
pk18mobsacB-RSP0847(D56A)	*R. sphaeroides* genomic regions containing the *RSP0847*D56A allele and genomic regions flanking the gene Gibson assembled into pk18; Km^R; for allelic exchange within RSP0847	This work
pk18mobsacB-RSP0847(D56E)	*R. sphaeroides* genomic regions containing the *RSP0847*D56E allele and genomic regions flanking the gene Gibson assembled into pk18; Km^R; for allelic exchange within RSP0847	This work
$pIND5_{spec}$	Replicable plasmid used for protein expression in *Rb. sphaeroides*.	[2]
pIND5spec-0847	Ectopic expression of *RSP0847* (*cenR*)	This work
pVP302K-N/C	Protein expression vector; Km^R lac promoter lacI, Tev site *rtxA* (*V. cholera*) coding sequence for 8×His-tag	[3]
pVP302K-N-$RSP1056_{cyto}$	pVP302K containing truncated *RSP1056* at M195	This work
pVP302K-C-RSP0847	pVP302K containing *RSP0847*	This work
pVP302K-C-RSP0847(D56A)	pVP302K containing *RSP0847* variant D56A	This work
pVP302K-C-RSP1274	pVP302K containing *RSP1274*	This work
pVP302K-C-RSP1083	pVP302K containing *RSP1083*	This work
pVP302K-N-0847D56E	pVP302K containing *RSP0847* variant D56E with N-terminal H_8 tag	This work
pVP302K-N-0847	pVP302K containing *RSP0847* with N-terminal H_8 tag	This work

C. Strains

Strains	Relevant characteristics	Reference
***Rhodobacter sphaeroides* strains**		
2.4.1	Wild-type strain	ATCC 17023
BDL021	Δ*RSP1056* (Δ*cenK*)	This work
BDL072	*RSP0847*D56A (*cenR* D56A)	This work
BDL019	*RSP0847*D56E (*cenR* D56E)	This work
BDL020	*RSP0847*D56E Δ*RSP1056* (*cenR* D56E Δ*cenK*)	This work
BDL013	$pIND5_{spec}$-*cenR*	This work
BDL121	$pIND5_{spec}$-*cenR* Δ*cenR*	This work
***Escherichia coli* strains**		
DH5α	F- Φ80*lac*ZΔM15 Δ(*lacZYA-argF*) U169 *rec*A1 *end*A1 *hsd*R17 (rK–, mK+) *phoA supE*44 λ– *thi*-1 *gyr*A96 *rel*A1	New England Biolabs
S17-1	*TpR SmR recA1 thiE1 pro-82 hsdR17 RP4-2-Tc::Mu-Km::Tn7* λpir	[4]
NEB 5-alpha competent *E. coli*	*fhu*A2 Δ(*argF-lacZ*)U169 *phoA gln*V44 Φ80 Δ(*lacZ*)M15 *gyr*A96 *rec*A1 *rel*A1 *end*A1 thi-1 *hsd*R17	New England Biolabs
B834	F- *hsdS metE gal ompT*	[5,6]
BL21 (DE3) pLysS CodonPlus	E. coli str. B F– *ompT gal dcm lon hsdSB*(rB– mB–) λ(DE3 [*lacI* lacUV5-T7p07 *ind1 sam7 nin5*]) [*malB*+]K-12(λS) pLysS[T7p20 orip15A](CmR) argU (AGA, AGG), ileY (AUA), leuW (CUA)	Novagen

References.

1. Schäfer A, Tauch A, Jäger W, Kalinowski J, Thierbach G, Pühler A. Small mobilizable multi-purpose cloning vectors derived from the Escherichia coli plasmids pK18 and pK19: selection of defined deletions in the chromosome of Corynebacterium glutamicum. Gene. 1994;145(1):69–73. doi: 10.1016/0378-1119(94)90324-7.

2. Ind AC, Porter SL, Brown MT, Byles ED, de Beyer JA, Godfrey SA, Armitage JP. Inducible Expression Plasmid for *Rhodobacter sphaeroides* and *Paracoccus denitrificans*. Appl. Environ. Microbiol. 2009;75:6613–6615. doi: 10.1128/AEM.01587-09.

3. Gall DL, Ralph J, Donohue TJ, Noguera DR. A Group of Sequence-Related Sphingomonad Enzymes Catalyzes Cleavage of β-Aryl Ether Linkages in Lignin β-Guaiacyl and β-Syringyl Ether Dimers. Environ Sci Technol. 2014;48(20):12454–63. doi: 10.1021/es503886d.

4. Simon R, Priefer U, Pühler AA. A broad range mobilization system for in vivo genetic engineering: transposon mutagenesis in Gram-negative bacteria. Nat Biotechnol. 1983;(1):784-791. doi: 10.1038/nbt1183-784.

5. Doherty AJ, Ashford SR, Brannigan JA, Wigley DB. A superior host strain for the over-expression of cloned genes using the T7 promoter based vectors. Nucleic Acids Res. 1995 Jun 11;23(11):2074-5. doi: 10.1093/nar/23.11.2074.

6. Wood WB. Host specificity of DNA produced by Escherichia coli: bacterial mutations affecting the restriction and modification of DNA. J Mol Biol. 1966 Mar;16(1):118-33. doi: 10.1016/s0022-2836(66)80267-x.
