# Supplementary material for: The essential Rhodobacter sphaeroides CenKR two-component system regulates cell division and envelope biosynthesis
Source: PLoS Genet. 2022 Jun 29;18(6):e1010270. doi: 10.1371/journal.pgen.1010270 (PMC9275681; doi:10.1371/journal.pgen.1010270)
Supplement: S3 Table — (PDF) [file pgen.1010270.s009.pdf]

Table S3. Genbank accession numbers for all protein sequences used in CenK/R phylogeny (Fig 6).

Organism	CenK homolog	CenR homolog
Rhodobacter sphaeroides 2.4.1	ABA80240.1	ABA80028.1
Rhodobacter ovatus	WP_097029852.1	WP_097029114.1
Cereibacter changlensis	PZX58573.1	WP_107663473.1
Defluviimonas denitrificans	WP_105515131.1	WP_105516383.1
Pseudorhodobacter aquimaris	WP_083444869.1	WP_050526965.1
Pseudorhodobacter wandonensis	WP_050524403.1	WP_050523141.1
Roseibacterium elongatum	WP_025311819.1	WP_025311137.1
Paracoccus aminovorans	WP_062561073.1	WP_074969281.1
Rhodobacter capsulatus	WP_055211899.1	WP_136906601.1
Roseicitreum antarcticum	WP_092887326.1	WP_092887229.1
Gemmobacter megaterium	WP_083701327.1	WP_076533744.1
Meinhardsimonia_xiamenensis	WP_092500460.	WP_092497609.1
Pararhodobacter aggregans	WP_107753170.1	WP_107752404.1
Paracoccus zeaxanthinifaciens	WP_022708257.1	WP_022707883.1
Pontivivens insulae	WP_108783177.1	WP_108783392.1
Oceanicella actignis	WP_211356277.1	WP_072746865.1
Maricaulis maris	WP_121209738.1	WP_041637067.1
Hirschia maritima	WP_155826066.1	WP_018997296.1
Hyphomonas oceanitis	WP_051624558.1	WP_035538430.1
Ponticaulis koreensis	WP_022695910.1	WP_022693572.1
Henriciella algicola	WP_119455183.1	WP_119453332.1
Bradyrhizobium erythrophlei	WP_074272850.1	WP_072818367.1
Parvularculaceae bacterium	MCB2096247.1	MAW81539.1
Amphiplicatus metriothermophilus	WP_089410642.1	WP_089410979.1
Marinicaulis flavus	WP_104828836.1	WP_104829420.1
Caulobacter crescentus (vibrioides)	WP_010918418.1	WP_024266002.1
Caulobacter segnis	WP_013077599.1	PZR32001.1
Caulobacter henricii	WP_035045754.1	WP_062151853.1
Phenylobacterium haematophilum	WP_183772596.1	WP_183775692.1
Asticcacaulis endophyticus	GGZ27123.1	WP_189484874.1
Brevundimonas bacteroides	WP_029414873.1	WP_029417475.1
Oceanicaulis alexandrii	WP_022701438.1	WP_009802422.1
Ruegeria atlantica	NOD30888.1	WP_058279748.1
Ruegeria litorea	WP_085794123.1	WP_085797046.1 (*sp.*)
Ruegeria pomeroyi	WP_011046299.1	WP_011049109.1
Thalassobius activus	WP_058313840.1	WP_209358005.1
Roseobacter cerasinus	WP_159974535.1	WP_159975066.1
Roseobacter denitrificans	WP_011567720.1	WP_011566403.1 (*sp.*)
Sulfitobacter pontiacus	WP_064215825.1	WP_005850360.1
Lutimaribacter pacificus	WP_149789044.1	WP_149788025.1
Roseovarius aestuarii	WP_085800950.1	WP_085800306.1
Nitratireductor aquibiodomus	WP_065816343.1	WP_065816950.1
Nitratireductor pacificus	WP_040676576.1	WP_008596599.1
Hoeflea phototrophica	WP_007199885.1	WP_007199422.1
Phyllobacterium phragmitis	WP_105743815.1	WP_105741337.1
Phyllobacterium leguminum	WP_110752236.1	WP_110749900.1
Agrobacterium tumefaciens	KIQ02346.1	AMD60228.1
Sinorhizobium meliloti	RVJ02864.1	WP_100672725.1
Chelativorans alearense	WP_163266599.1	WP_163265216.1

Pseudovibrio denitrificans	WP_054784296.1	WP_041767864.1
Azorhizobium caulinodans	WP_043879669.1	WP_012168621.1
Bosea robiniae	WP_091856047.1	WP_061966680.1
Pararhizobium polonicum	WP_099050751.1	WP_068955266.1
Mesorhizobium albiziae	WP_188130341.1	WP_149759750.1
Neorhizobium galegae	WP_046664845.1	WP_210105920.1
Brucella rhizophere	WP_094579191.1	WP_094578402.1
Brucella abortus	WP_002963438.1	WP_002965068.1
Brucella melitensis	SPU63212.1	WP_070997026.1
Labrenzia (*Roseibium*) *alba*	WP_055675062.1	WP_055677803.1
Labrenzia (*Roseibium*) *aggregate*	WP_206904135.1	WP_190291169.1
Nitrobacter winogradskyi	WP_141383471.1	WP_011313424.1
Nitrobacter hamburgensis	WP_011512071.1	WP_011508702.1
Rhodopseudomonas palustris	WP_044412660.1	MBI5130782.1
Sneathiella sp.	PHQ71087.1	PHQ71627.1
Sneathiella litorea	MZR29347.1	WP_161314357.1
Sneathiella chinensis	WP_169559456.1	WP_169559000.1
Kiloniella majae	WP_085900939.1	WP_047764063.1
Kiliella laminaria	WP_020590891.1	WP_020590402.1
Skermanella rosea	WP_203096208.1	WP_203098182.1
Denitrobaculum tricleocarpae	WP_142894422.1	WP_142896990.1
Fodinicurvata sediminis	WP_022727682.1	WP_022727791.1
Roseospirillum parvum	WP_092616041.1	WP_092621908.1
Novispirillum itersonii	WP_184264282.1	WP_184262290.1
Ferruginivarius sediminum	WP_114583718.1	WP_114580497.1
Azospirillum halopraeferens	WP_029009133.1	WP_029010793.1
Azospirillum lipoferum	WP_085554914.1	WP_085555599.1
Thalassospira profundimaris	WP_114112327.1	WP_114100359.1
Thalassospira povalilytica	WP_206926285.1	WP_062950084.1 (*sp.*)
Thalassospira xiamenensis	WP_097053219.1	WP_101246657.1 (*sp.*)
Curvivirga aplysinae	WP_155157144.1	WP_155154090.1
Aestuariispira insulae	WP_115938537.1	WP_115936241.1
Oceanibaculum pacificum	WP_067557655.1	WP_067551072.1
