# Supplementary material for: The essential Rhodobacter sphaeroides CenKR two-component system regulates cell division and envelope biosynthesis
Source: PLoS Genet. 2022 Jun 29;18(6):e1010270. doi: 10.1371/journal.pgen.1010270 (PMC9275681; doi:10.1371/journal.pgen.1010270)
Supplement: S4 Table — (PDF) [file pgen.1010270.s010.pdf]

Table S4. PatSer motif search (Fig 6) and PWM for CenR binding motif in *Rhodobacter sphaeroides* 2.4.1

A. PatSer motif search

Identified gene[1]	Position (distance from start codon)[2]	Score[3]	ln(p-value)	Sequence
***Rhodobacter sphaeroides* 2.4.1**				
cenR	183	7.4	-9.45	TTGCAGGAAATTGAA
msrA	199	9.98	-12.6	TGGCGCGAGCGTGAT
RSP1807	231	8.59	-10.77	TCGCAAAACCTTGAT
RSP1860	177	7.11	-9.16	TGGCAAAAATGTCAC
surA	142	9.01	-11.28	TGACATGATCGTGTG
tolQ	213	9.98	-12.6	TGACGCAGATGTGTT
***Agrobacterium tumefaciens* BIM B-1315G**				
cenR (IAI05_12475)	65	8.44	-10.6	TCACGAGATTTTGCT
msrA (IAI05_13425)	101	8.46	-10.62	TGGCGCTTCATTGAA
RSP1807 (IAI05_12080)	197	10.11	-12.79	TGACTTTCTATTGAA
RSP1860 (IAI05_01710)	171	9.5	-11.91	ACACATTAACGTGAA
surA (IAI05_17235)	239	3.26	-5.89	TGGAAGTTTTTTCAG
tolQ (IAI05_18005)	240	7.88	-9.96	TAACACGCGTTTGAA
***Brucella abortus* 2308**				
cenR (BAB1_2006)	121	4.33	-6.68	TCAAGGTCTTTCGAG
msrA (BAB2_1029)	116	7.5	-9.55	AGAAGCGACCGTGAC
RSP1807 (BAB1_0088)	146	7.66	-9.73	TGAATTCCTATTGAT
RSP1860 (BAB1_0408)	94	5.39	-7.55	TAGCAATGGTCTGAT
surA (BAB1_1944)	160	7.32	-9.37	TTGCTTCAAAGTGAT
tolQ (BAB1_1712)	238	5.9	-8	TAACGGATTTGCGAT
***Caulobacter crescentus* NA1000**				
cenR (*CCNA_03859*)	195	4.02	-6.44	TCCCGCAAGCTCGAA
msrA (*CCNA_01046*)	65	3.7	-6.21	AGAGAGGGATTCGAA
RSP1807 (*CCNA_03780*)	259	8.04	-10.14	TCACGGCCTCGTGAA
RSP1860 (*CCNA_03062*)	125	6.32	-8.39	TCAAGTGCTTGCGAG
surA (*CCNA_03163*)	145	3.6	-6.13	TGGTGTCAACGCGAA
tolQ (CCNA_03341)	66	2.49	-5.36	TGAGGTGCTGTTCGA
***Rhodopseudomonas palustris* RCB100**				

cenR (RPA0283)	55	5.73	-7.85	ACAATCGCGCGTGAT
msrA (RPA4834)	100	7.19	-9.02	TGAAATTGAGTTGAG
RSP1807 (RPA0201)	108	5.82	-7.93	TGAGTGGCAGGTGAA
RSP1860 (RPA0201)	NA	NA	NA	NA
surA (RPA0511)	83	6.60	-8.65	TTTCATCATTTTGAC
tolQ (RPA1117)	143	7.19	-9.24	TGACAGGGGATTGAG

[1]blastn search to identify homologs of each *Rb. sphaeroides* gene (locus tag listed).
[2]Identified in 300bp genomic region upstream of start codon using in search.
[3]Scores determined by comparison of the base position in the sequence to the same position in the PWM (below). Scores for each position are summed for the length of the sequence, larger score means more positions matched more frequent bases in the PWM.
NA - could not identify a homolog with high sequence homology.

B. PWM CenR binding motif in *Rhodobacter sphaeroides* 2.4.1 (Fig 4A) formatted for Patser

**nucleotide**	**Logo Position**														
	**1**	**2**	**3**	**4**	**5**	**6**	**7**	**8**	**9**	**10**	**11**	**12**	**13**	**14**	**15**
**A**	1	0	7	1	2	2	2	3	2	1	0	0	0	8	3
**C**	0	3	0	8	0	4	1	2	2	4	1	1	0	0	4
**G**	0	6	3	1	6	1	5	3	3	1	6	0	10	1	1
**T**	9	1	0	0	1	3	2	2	3	4	2	9	0	1	3
